# Scene statistics and noise determine the relative arrangement of receptive field mosaics

**DOI:** 10.1073/pnas.2105115118

**Published:** 2021-09-23

**Authors:** Na Young Jun, Greg D. Field, John Pearson

**Affiliations:** ^a^Department of Neurobiology, Duke University, Durham, NC 27708;; ^b^Department of Biostatistics & Bioinformatics, Duke University, Durham, NC 27708

**Keywords:** retina, mosaic, computational model, efficient coding

## Abstract

Many sensory systems utilize parallel ON and OFF pathways that signal stimulus increments and decrements, respectively. These pathways consist of ensembles or grids of ON and OFF detectors spanning sensory space. Yet, encoding by opponent pathways raises a question: How should grids of ON and OFF detectors be arranged to optimally encode natural stimuli? We investigated this question using a model of the retina guided by efficient coding theory. Specifically, we optimized spatial receptive fields and contrast response functions to encode natural images given noise and constrained firing rates. We find that the optimal arrangement of ON and OFF receptive fields exhibits a transition between aligned and antialigned grids. The preferred phase depends on detector noise and the statistical structure of the natural stimuli. These results reveal that noise and stimulus statistics produce qualitative shifts in neural coding strategies and provide theoretical predictions for the configuration of opponent pathways in the nervous system.

## Significance

Across a wide variety of species, cells in the retina specialized for signaling either increases (ON) or decreases (OFF) in light represent one of the most basic building blocks of visual computation. These cells coordinate to form mosaics, with each cell responsible for a small, minimally overlapping portion of visual space, but the ways in which these mosaics could be spatially coordinated with each other are relatively unknown. Here, we show how efficient coding theory, which hypothesizes that the nervous system minimizes the amount of redundant information it encodes, can predict the relative spatial arrangement of ON and OFF mosaics. The most information-efficient arrangements are determined both by levels of noise in the system and the statistics of natural images.

Across many sensory systems, neurons encode information about either increments or decrements of stimuli in the environment, so-called ON and OFF signals. This division between ON and OFF signaling has been observed in visual (1, 2), thermosensory (3), auditory (4), olfactory (5), and electrosensory (6) systems. This organization has the advantage that neurons can be tasked with signaling increments or decrements in steady-state stimulus levels with fewer spikes, thereby resulting in more efficient neural codes (7, 8). Moreover, when the number of potential stimuli is large, neurons often specialize; for example, they only respond to a small region of visual space or a narrow auditory frequency band. The combination of these coding strategies raises two questions. First, how should a particular set of detectors, either the ON or OFF cells, arrange themselves most efficiently to cover stimulus space? Second, what is the optimal relative arrangement of ON and OFF detector grids? For one system, the retina, the answer to the first question is clear from previous work: Detectors of a particular type tile stimulus space and exhibit overlap near the 1-sigma boundary of a Gaussian receptive field (9–13). The answer to the second question, what might be called the “sensor alignment problem,” has received comparatively little attention and is the focus of this study.

Conceptually, there are three general possibilities for how the sensor alignment problem could be solved. One possibility is that the grids of sensors are statistically independent, meaning the locations of receptive fields in one grid provide no information about the receptive field locations in the other grid. A second possibility is that the two grids are aligned, meaning the receptive field centers in one grid are closer than expected by chance. The third possibility is that the two grids are antialigned, meaning the receptive field centers in the two grids are further apart than expected by chance. On general information theory grounds, the optimal solution is likely to depend on noise in the encoding process and the statistics of the encoded stimuli (14, 15).

While most anatomical studies of retinal mosaics indicate they are statistically independent (16–18, but see ref. 19), we have recently shown that grids of ON and OFF receptive field (henceforth called “mosaics”) formed by retinal ganglion cells (RGCs) are antialigned when those cells encode similar visual features (20). Here, we show how these results can be explained through the lens of efficient coding theory (7). This theory argues that sensory systems should aim to reduce the redundancy present in sensory input while minimizing metabolic costs, thereby reliably encoding natural stimuli with fewer spikes. Efficient coding theory has been successful at explaining many aspects of sensory processing and retinal physiology, including center-surround receptive fields, the formation of mosaics, and a greater proportion of OFF than ON cells (7, 11, 15, 21, 22). Thus, we asked whether efficient coding theory might predict the optimal spatial arrangement of ON and OFF receptive field mosaics within the retina. Our approach to this question involved optimizing a model that approximates the processing performed by many RGCs (21). By maximizing the mutual information between an (input) library of natural images and (output) spike rates, we examined the effects of image statistics and encoding noise on the optimal arrangement of ON and OFF mosaics.

In this model, we found that the optimal spatial arrangement was a pair of approximately hexagonal mosaics of ON and OFF receptive fields. However, surprisingly, the relative alignment of these mosaics depended on the input noise, output noise, and the statistics of the natural image set. When output noise was low, the mosaics were aligned, with ON and OFF receptive fields centered at nearby locations more often than expected by chance. When output noise was relatively high, antialignment became the favored arrangement. Surprisingly, the content of the image set also strongly influenced the transition between aligned and antialigned mosaics. In particular, when image sets contained more “outlier” images with particularly large luminance or contrast values, antialignment became the favored state for fixed input and output noise. We demonstrate analytically and confirm computationally that as noise parameters or stimulus statistics vary, mutual information changes smoothly, while the optimal mosaic arrangements undergo a sudden, qualitative shift. Finally, we confirm these predictions by showing that systematic manipulations of the training dataset change the phase boundary in a manner predicted by an analytical model. These findings underscore the crucial role played by both noise and the statistics of natural stimuli for understanding specialization and coordination in sensory processing.

## Methods

### Linear–Nonlinear Efficient Coding Model.

The model used to examine efficient coding is based on previous work (21), designed to find the optimal encoding of visual scenes. The model takes patches of natural scenes (23) as the input, each of which has 18×18 (324) pixels. Each input image was multiplied by a circular mask to avoid cases in which corners in the optimization region produce corner artifacts that dominate the final configuration. The circular masking effectively left about 255 pixels in each input image patch. The model consisted of a linear–nonlinear architecture with learned linear filters and nonlinearities. Given an input stimulus x, an input noise $n_{x}$, and the linear filter $w_{j}$ for each neuron j, the nonlinearities were modeled as softplus functions of the linearly filtered noisy stimulus for each neuron $y_{j}=w_{j}^{⊤}\left(x+n_{x}\right)$:

$$\eta \left(y_{j}\right)=\log\left(1+\exp\left(\beta \cdot y_{j}\right)\right)/\beta$$ [1]

using a fixed value of β=2.5. We empirically chose a fixed value of β=2.5 that produced a stable optimization trajectory. The output of the model consisted of 100 units that mimic RGCs, whose firing rates are modeled using neuron-specific gain and threshold parameters $\gamma_{j}$ and $\theta_{j}$, plus an additive output noise $n_{r,j}$:

$$r_{j}\left(y_{j}\right)=\gamma_{j}\cdot \eta \left(y_{j}-\theta_{j}\right)+n_{r,j}.$$ [2]

Note that scaling r, γ, and $n_{r}$ by the same factor leaves the model invariant, so we choose units in which the mean firing rate is Er=1. We used a first-order approximation that treats the conditional distribution of activation as a Gaussian, which allowed us to derive a closed-form expression for the mutual information. A detailed derivation is provided in *SI Appendix*. The model was trained to maximize the mutual information between the input images and the firing rates of the output neurons. To model the metabolic cost of spiking, the average firing rate of each neuron across images was constrained to 1, which was enforced via an augmented Lagrangian method with quadratic penalty. Input and output noise were modeled as independent and identically distributed Gaussian noise (zero means and SDs of $\sigma_{\text{in}}$ and $\sigma_{\text{out}}$, respectively).

**Fig. 1.**
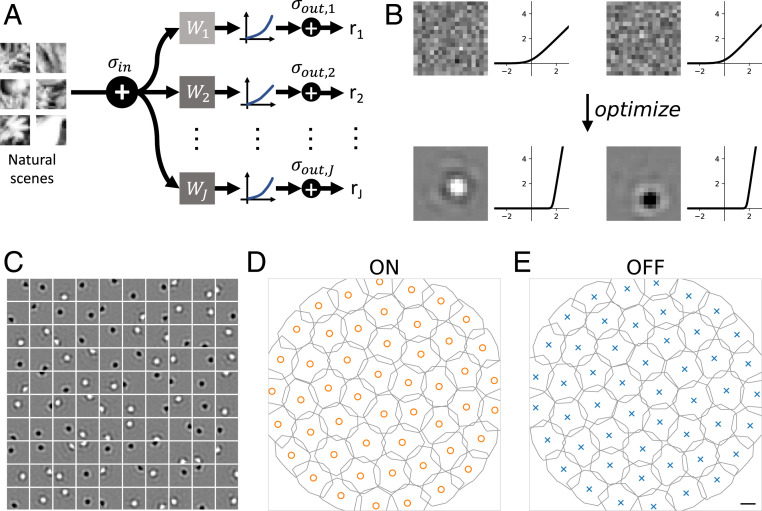
Spatial tiling of receptive fields is predicted by efficient coding. (*A*) Efficient coding model architecture. Images of natural scenes (plus input noise $\sigma_{\text{in}}$) are multiplied by linear filters $w_{j}$, passed through a nonlinear function $\eta_{j}$, and perturbed by output noise $\sigma_{\text{out}}$, resulting in a firing rate $r_{j}$ for neuron j. (*B*) Examples of initial and optimized filters and nonlinearities, where the filters are initialized from a white noise distribution and converge to ON and OFF kernels with a center-surround pattern. The nonlinearities are initialized as unscaled (but slightly perturbed) softplus functions and converge to nonzero threshold values. (*C*) A plot of 100 kernels from a trained model with $\sigma_{\text{in}}$ = 0.4, $\sigma_{\text{out}}$ = 3.0. (*D* and *E*) Contour plot showing the tiling of the ON and OFF kernels. The contours of the two types of kernels are drawn where the normalized pixel intensity is ± 0.21. Orange circles and blue x’s indicate receptive field centers of mass for ON and OFF cells, respectively. Scale bar is width of one image pixel.

The parameters of the model were the receptive field filter weights, the gain of the nonlinearity, and the nonlinearity threshold of each output RGC. To preserve positive gain for each output unit, we optimized the logarithms of the corresponding parameters. Filter weights were initialized by drawing samples from a Gaussian distribution (mean zero, SD 1; see Fig. 1*B*, *Top*). The parameters of the nonlinearities (log gain and threshold) were initialized by sampling from a uniform distribution [0, 1]. The parameters were then optimized using stochastic gradient descent with a learning rate of 0.001. During training, we calculated gradients using minibatches of 100 image patches at a time, renormalizing the filters to have unit norm after each gradient step. Models were trained for 1 million iterations, which we found sufficient in practice to ensure convergence of the mutual information. As a means of escaping local optima, between 200,000 and 500,000 iterations, two modifications were applied to the optimization procedure, which we call jittering and centering. These were used to speed convergence. The jittering operation was applied every 5,000 iterations and consisted of raising each element of the kernels to the power 1.25; this makes high-amplitude portions of the filters more pronounced while attenuating low-amplitude portions. The centering operation penalized the spatial spread of the kernels by adding the mean of the spatial variance of each kernel as an additional loss term; this encourages the kernels to be localized around their centers. These two operations allowed kernels that were trying to “squeeze” into an already formed mosaic the space to do so, effectively speeding the optimization and allowing all kernels to contribute to the encoding. After these operations, the model was optimized without the modifications for another 500,000 iterations. We confirmed that jittering and centering do not alter the final converged shape of the kernels.

### Median Nearest-Neighbor Analysis of the Spatial Relationships between the Mosaics.

**Fig. 2.**
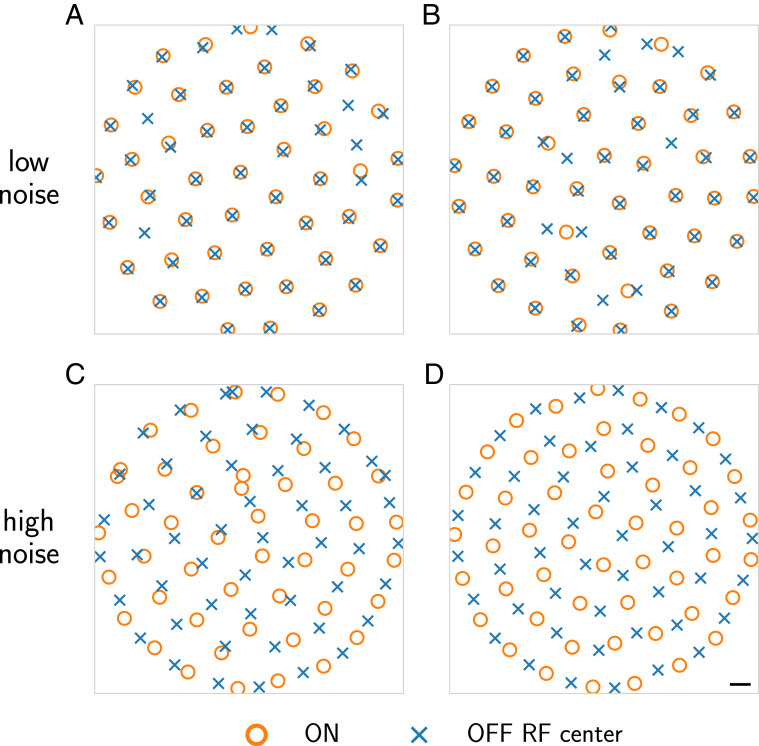
Spatial coordination of receptive field mosaics depends on input and output noise level. Receptive field centers for ON (orange circle) and OFF (blue x) cells under differing sets of noise parameters. (*A*) ($\sigma_{\text{in}}$ = 0.02, $\sigma_{\text{out}}$ = 1.0), (*B*) ($\sigma_{\text{in}}$ = 0.1, $\sigma_{\text{out}}$ = 1.0), (*C*) ($\sigma_{\text{in}}$ = 0.4, $\sigma_{\text{out}}$ = 2.0), (*D*) ($\sigma_{\text{in}}$ = 0.4, $\sigma_{\text{out}}$ = 3.0). The first two parameter sets result in aligned mosaics, while the latter two, at higher levels of output noise, are antialigned. Scale bar is width of one image pixel.

To quantify the relative alignment of ON and OFF mosaics, we used a metric based on the median nearest-neighbor distances between heterotypic receptive field centers. For each receptive field center, we found the nearest center with the opposite polarity to define its heterotypic nearest neighbor distance and calculated the median of these values (see Eq. **3**). When ON and OFF mosaics are nearly aligned, this value is close to zero, as in Fig. 2 *A* and *B*:

$$\begin{array}{c} \text{Median}\\ \text{NN distance} \end{array}=\underset{c\in \text{RF centers}}{\text{median}}\left(\underset{c^{\prime }\in \begin{array}{c} \text{RF centers}\\ \text{with opposite}\\ \text{polarity to c} \end{array}}{\min}\|c-c^{\prime }\|\right).$$ [3]

### One-Shape Model for Rapid Exploration of Mosaic Arrangements.

**Fig. 3.**
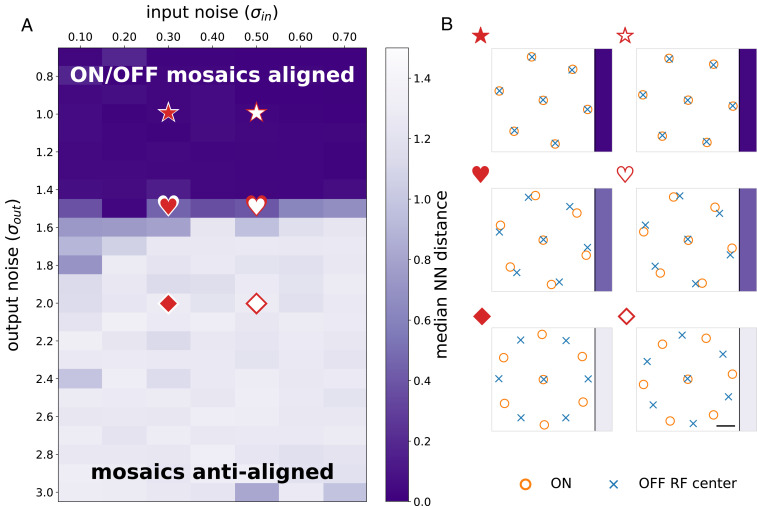
Optimal mosaic configurations transition from aligned to antialigned as a function of noise. (*A*) As output noise increases, optimal mosaic configurations shift from aligned to antialigned, and this holds over a range of input noise values. Color indicates median nearest neighbor distance, with darker indicating more clearly aligned (see Eq. **3** and *Methods*). The existence of a phase boundary between the two arrangements is clear. (*B*) Examples of optimal mosaic arrangements for representative input and output noise combinations. Symbols denote the corresponding locations in *A*. Note the existence of optimal configurations between aligned and fully antialigned (hearts) for some parameter values. Scale bar is width of one image pixel.

The full 18 × 18 system required many hours to optimize, motivating the development of a smaller, scaled-down system for exploring how mosaic arrangements depended on different noise parameters (Fig. 3) or different stimulus statistics (see Fig. 6). Here, we restricted input images to 7 × 7, fixed the number of ON and OFF cells at 7 each (14 total), and adopted a fixed parametric form for the receptive field. Thus, the optimization only learned receptive field center locations and a small number of receptive field shape parameters. The receptive field function was parameterized as a difference of two Gaussian curves, with a third parameter determining their relative magnitude:

$$\kappa \left(r\right)\propto e^{-ar^{2}}-ce^{-br^{2}},\;a,b,c>0.$$ [4]

Optimization in this setup involves fewer parameters, as well as allowing us to use more efficient first-order optimization methods like Adam (24). To examine the dependence of mosaic arrangement on noise, a grid search was performed over combinations of input and output noise values, with input noise ($\sigma_{\text{in}}$) ranging from 0.0 to 0.75 and output noise ($\sigma_{\text{out}}$) from 0.75 to 3.0. As previously argued (21), these values lie within the physiological range for naturalistic stimuli. The optimization was run three times for every pair of noise values, and the model resulting in the highest mutual information was retained (Fig. 3). This model was also used to examine the dependence of mosaic alignment on the statistics of natural scenes (see Fig. 6). In these simulations, the input noise was held fixed while the output noise and distribution of natural images were varied to examine the interaction between these factors on the resulting mosaic arrangements.

## Results

### Linear–Nonlinear Efficient Coding Model of the Retina.

We asked how mosaics of ON and OFF cells should be arranged to efficiently encode natural scenes. To answer this question, we trained a model of retinal processing to maximize the mutual information between a library of natural images and a set of output firing rates (Fig. 1*A*). The model consists of a single layer of linear–nonlinear units intended to approximate the encoding performed by RGCs (10, 11, 21, 25–27). These model cells linearly filter the input stimulus weighted by a spatial mask or “receptive field” and sum the resulting values. This number, loosely analogous to a membrane potential, is then fed through a nonlinearity to approximate rectified neural firing rates. The model also included two sources of noise: 1) a pixelwise input noise, which models stochasticity in phototransduction, and 2) an output noise, which models stochasticity in output firing rates. A key difference between the two is that the input noise is subject to the nonlinearity, and thus its effect becomes stimulus-dependent, while the output noise is not. We trained the model by optimizing mutual information under the constraint of a fixed mean activation (across all images) for each neuron (see *Methods*). This constraint is intended to model the metabolic cost of generating action potentials.

### Receptive Field Tiling Is Predicted by Efficient Coding.

Following training, units in the model exhibited either ON-center or OFF-center receptive fields with strongly rectified nonlinearities (Fig. 1 *B* and *C*). Each population of ON-center units and OFF-center units (referred henceforth as ON cells and OFF cells, respectively) exhibited a mosaic-like organization: their receptive fields tiled space (Fig. 1*D*) (21). When varying noise levels, we reliably found circular ON and OFF receptive fields with input noise between 0.00 and 0.75 and output noise between 0.75 and 3.00. Input and output noise levels outside these ranges produced irregular receptive field shapes (*SI Appendix*, Fig. S1), as did nonnatural distributions of images (*SI Appendix*, Fig. S7). Therefore, we restricted the analysis to this range of noise regimes and focused on natural image patches.

### Mosaic Arrangements Depend on Input and Output Noise.

We next analyzed how these optimized mosaics of ON and OFF receptive fields were spatially arranged with respect to one another. Each optimized mosaic formed a nearly hexagonal grid (Fig. 1*D*). Analysis of the spatial relationships between the mosaics in Fig. 1 *D* and *E* revealed they were antialigned (Fig. 2*D*). However, as we explored the effect of changing the amounts of input and output noise on the optimization of this model, we observed other mosaic combinations that were aligned (e.g., Fig. 2*D*). Generally, small input and output noise values yielded aligned mosaics, while larger values produced antialigned mosaics (Fig. 2 *A*–*D*). Importantly, for a fixed amount of input and output noise, multiple instances of the optimization produced consistent results, indicating that mosaic alignment and antialignment depended on the specific amount of input and output noise, and did not depend on different initializations to the optimization. Moreover, these same effects held when image patches were twice as large (*SI Appendix*, Fig. S8), arguing against simple edge effects. These results motivated a more thorough analysis of how the relative spatial organization between the two mosaics depended on input and output noise.

### A Simplified “One-Shape” Model Captures the Noise Dependence of Mosaic Arrangements.

To examine the dependence of the mosaic arrangements on noise over a range of densely sampled values we utilized a simpler “one-shape” model that trained much more quickly than the full model (see *Methods*). This model assumed that all receptive fields shared a common shape (a difference of concentric Gaussians), while optimizing over both the parameters of this shape and the locations of the receptive fields (see *Methods*). The learned kernels of this simplified model also exhibited ON and OFF receptive fields (*SI Appendix*, Fig. S2*A*) and ON and OFF mosaics (*SI Appendix*, Fig. S2*B*). In addition, the optimal radial kernel exhibited a center-surround organization (*SI Appendix*, Fig. S2*C*). To further speed training, we also reduced the number of units in the model to seven ON and seven OFF cells (their polarities were fixed during the optimization). These simplifications allowed us to rapidly judge if the optimization preferred alignment or antialignment (Fig. 3). To ensure these simplifications did not strongly bias the results, we compared the mosaic arrangements for particular input and output noise combinations across the one-shape model and the full model. In all cases examined, the two models produced matching aligned or antialigned results (*SI Appendix*, Fig. S3). Thus, the one-shape model is a useful proxy for larger-scale and more general optimizations and could be used to rapidly and reliably determine if mosaics were aligned or antialigned for a given set of noise parameters.

### Mosaics Exhibit a Phase Change as a Function of Input and Output Noise.

To determine the optimal arrangement of ON and OFF mosaics for different amounts of input and output noise, we performed a grid search over input and output noise values using the one-shape model. We ran the model with input noise ranging from 0.00 to 0.75 in steps of 0.05 and output noise from 0.75 to 3.00 in steps of 0.10. Optimizing the location of seven receptive field centers in a circular space nearly always resulted in a single kernel in the center surrounded by the remaining six kernels at the vertices of a hexagon (see Fig. 3). When the output noise level was low, ON and OFF receptive field mosaics were aligned as in the first row of Fig. 3*B* and resulted in near-zero median nearest-neighbor distances (dark purple colored area in Fig. 3*A*), whereas ON and OFF mosaics were antialigned under higher output noise levels as in the last row of Fig. 3*B*, resulting in larger median nearest-neighbor distances (lighter colored area in Fig. 3*A*). In particular, this analysis reveals an abrupt transition between mosaic alignment and antialignment (Fig. 3*A*). Thus, our model optimization indicates the solution to the sensor alignment problem depends on noise present in the encoding process. Below, we build an analytical model for understanding this result.

### Retinal Phase Transitions from an Analytic Model of Efficient Coding.

To understand the factors that give rise to the phase transition between aligned and antialigned mosaics, we developed a simplified one-dimensional analytic model in which ON and OFF grids have identical nonlinearities, fixed kernels, fixed spacing, and are only allowed to shift relative to one another (*SI Appendix*, *Supporting Information Text*). Moreover, we assume that images are drawn from a Gaussian distribution with a 1/f frequency spectrum that is the one-dimensional equivalent of the two-dimensional scale-free distribution of natural images (14, 28), though other frequency distributions produced the same effect (*SI Appendix*, Fig. S9). We verified via simulation that, even with all of these restrictions, the phase transition occurs just as in the less constrained models in both one and two dimensions, suggesting that the remaining free parameters—gain, threshold, and mosaic alignment—are sufficient to account for the phase transition (*SI Appendix*, Figs. S5 and S6).

To gain additional insight into the origins of the transition, we analyzed the behavior of this model in the more analytically tractable limit of near-independent neurons. The starting point for this analysis is a recognition that the conditional entropy between stimulus and neural response can be conveniently written as a sum of single-neuron terms and higher-order corrections:

$$\begin{array}{ll} I\left(X;R\right) & =H\left(X\right)-H\left(X|R\right)\\ & =const+2NI_{1}-N\left(N-1\right)h_{2}-N^{2}h_{2^{\prime }}+\cdots \;. \end{array}$$ [5]

Here, H(X) is the (constant) entropy of the stimulus, 2N is the number of ON + OFF cells, $I_{1}$ is the contribution of each neuron individually, and $h_{2}$ and $h_{2^{\prime }}$ are correction terms due to nonindependence (and thus redundancy) of ON–ON/OFF–OFF (same polarity) and ON–OFF (opposite polarity) cells, respectively (*SI Appendix*, *Supporting Information Text*, section B). Importantly, these corrections are always negative and vanish for widely separated cells. For our analysis, we focus only on these leading order terms, corresponding to an assumption of approximately independent neurons with small pairwise interactions. While this assumption is violated for natural images, which possess long-range correlations and induce triplet and higher-order interactions, our analytical results are nonetheless matched by simulations in the full model, suggesting that intuitions gleaned from this approximation are sufficient to explain the transition between alignment and antialignment. In the following two sections we show 1) that noise and image statistics set the optimal neuron response thresholds and 2) that these thresholds control the amount of redundancy in the population responses, thereby dictating whether mosaic alignment or antialignment is the preferred state.

### Output Noise and Image Outliers Drive Increases in Optimal Neuron Response Thresholds.

**Fig. 4.**
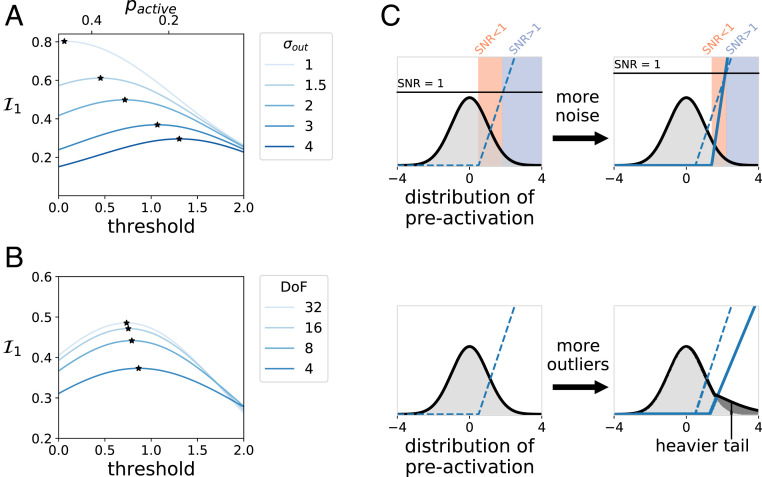
Optimal threshold increases with higher noise and heavier-tailed image distributions. (*A* and *B*) Contribution of a single neuron to the mutual information as a function of threshold. Stars mark optimal thresholds for multiple parameter values. (*A*) When the input distribution is Gaussian, the optimal threshold increases with higher output noise, even as overall information decreases. (*B*) When the input distribution is heavier-tailed, modeled with a Student’s *t* distributions with varying degrees of freedom, the optimal threshold again increases. (*C*) Schematic illustrating the effects of increased noise or outliers. With increased output noise, neurons’ SNR (black line, *Upper Right*) decreases, and efficient coding predicts that units should increase threshold and gain to reduce low SNR responses (peach). Similarly, when preactivations are heavy-tailed (*Lower Right*), efficient coding predicts that thresholds should increase and gains slightly decrease (*SI Appendix*, Fig. S11), since more mass is contained in outliers. Thus, both output noise and heavy tails lead to higher thresholds.

First, we considered optimizing only the $I_{1}$ term above, which captures the contribution of each neuron individually to encoding stimulus information. In particular, we focused on the influence of output noise and the distribution of natural images on the output nonlinearities of the neurons (we focused on output noise because the transition depended much more on output than input noise; see Fig. 3*A*):

$$I_{1}=p_{1}\log\left(1+\frac{g_{0}}{\sigma_{\text{in}}^{2}+\sigma_{\text{out}}^{2}\gamma^{-2}}\right),$$ [6]

where $g_{0}$ is a constant that depends on the image distribution and the receptive field shape, $p_{1}$ is the probability that the neuron is active, γ is the neuron’s gain, and these latter two are related through the constraint that the average response of the neuron is 1 (*SI Appendix*, Eq. **11**). As the output noise of neurons increases, maximizing $I_{1}$ dictates that neurons should respond by increasing their firing threshold (Fig. 4*A*). This allows the neurons to reserve spikes for stimuli with high signal-to-noise ratios (SNRs) (29). Thus, units respond less often but do so with higher gain when active, thereby mitigating the impact of noise. In addition, the optimal firing threshold depends on the nature of the image distribution. For neuron activation distributions with more outliers, thresholds increase as the tail of the distribution becomes heavier (Fig. 4*B*). Intuitively, this is because heavier-tailed distributions require higher thresholds to maintain the same overall response probability. Finally, it can be shown that decreasing the input noise has the effect of increasing the effective threshold (*SI Appendix*, *Supporting Information Text* and Eq. **12**). Thus, the optimal spiking threshold is determined both by the input and output noise and the distribution of natural scenes (schematized in Fig. 4*C*) (8).

### Mosaic Antialignment Achieves Redundancy Reduction at High Neuron Thresholds.

**Fig. 5.**
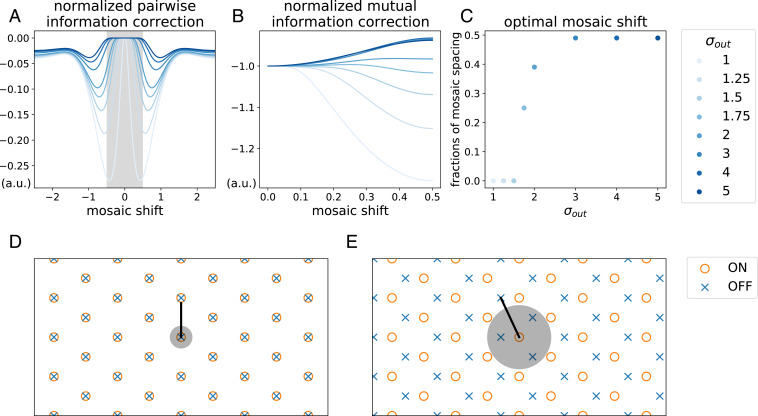
Pairwise mutual information corrections explain the phase transition between alignment and antialignment. (*A*) Normalized pairwise information correction ($-Nh_{2^{\prime }}/p_{1}^{2}$) for pairs of ON and OFF neurons as a function of mosaic shift. One unit is the spacing between nearest-neighbor receptive field centers of the same polarity. With low output noise, an aligned position (i.e., no shift) is the only placement to avoid a large negative information correction, while with higher output noise an ON–OFF pair can still have a finite shift within the gray area without incurring any penalty. (*B*) The total normalized pairwise information correction as a function of mosaic shift, assuming equispaced ON and OFF receptive field centers filling the one-dimensional space. With low output noise, any mosaic shift lowers mutual information, while with high output noise the pairwise mutual information becomes less negative when the mosaic is shifted by 0.5, i.e., when antialigned. (*C*) The optimal mosaic shift as a function of output noise. For $\sigma_{\text{out}}=1.7,\sigma_{\text{out}}=2$, the optimal mosaic shift lies between 0 (alignment) and 0.5 (antialignment), resembling the arrangements we obtained in the middle row of Fig. 3*B*. (*D*) Schematic of optimal two-dimensional mosaics in a low-noise regime. The gray circle indicates the area within which an OFF cell can be located with zero pairwise correction to mutual information. (*E*) Schematic of optimal two-dimensional mosaics in a high-noise regime. Here, the gray area has grown (as in *A*), allowing three neighbors of the opposite polarity to fit inside without loss in mutual information.

Next, we considered the effect of ON–OFF pair corrections to Eq. **5**. These terms, represented by $-N^{2}h_{2^{\prime }}$ above, capture the effects of redundancy in ON–OFF encoding: They are always negative and are the only contributions to mutual information that change as a function of alignment. Thus, while the overall trend is that mutual information decreases with increasing threshold, mosaic configurations may be more or less efficient for information transmission as they limit the impact of these terms. More specifically,

$$h_{2^{\prime }}=-\frac{1}{N^{2}}\underset{i,j}{\sum }p_{2}\left(d_{ij}\right)\log R\left(d_{ij}\right)\geq 0,$$ [7]

where $d_{ij}$ is the distance between ON cell i and OFF cell j, $p_{2}$ is the probability that the pair is coactive, and 0<R<1 is a function that is approximately independent of both output noise and neuron nonlinearity (*SI Appendix*, *Supporting Information Text*, section B). This correction term is small for large separations, large around the intramosaic spacing, and almost zero in a region around zero intercell distance (Fig. 5*A*). Importantly, the width of this “independence zone” around zero intercell distance widens as output noise grows larger. This is explained by the fact that as output noise increases so does the threshold (Fig. 4*A*), and at large threshold values nearby opposite polarity pairs are almost never coactive (*SI Appendix*, *Supporting Information Text*, section B).

This analysis gives rise to the following account of the phase transition: At low levels of output noise or light-tailed distributions of neuron activations, neuronal thresholds are low, and only aligned receptive fields avoid the large $N^{2}h_{2^{\prime }}$ penalty. Thus, in one dimension, each ON cell is nonredundant (save one bit of sign information; *SI Appendix*, *Supporting Information Text*, section C) with its aligned OFF partner, but image correlations make it highly redundant with the two OFF (and two ON) cells on either side. Likewise, in two dimensions, each ON cell is nonredundant (save one bit of sign information; *SI Appendix*, *Supporting Information Text*, section C) with its aligned OFF partner, but it suffers large negative corrections to encoding efficiency from its six other OFF neighbors. However, as activity thresholds rise, a finite-sized region develops around each cell within which other receptive fields of opposite polarity are almost never coactive (Fig. 5*A*, gray). This region of approximate independence grows with threshold, to the point that, when it is sufficiently large, more than one OFF cell can fit inside when the mosaics are antialigned (Fig. 5 *D* and *E*, gray circle). Thus, antialignment results in lower redundancy (via reduced $h_{2^{\prime }}$ penalties) and increased mutual information. This picture is borne out quantitatively by the model: As output noise (and thus threshold) increases, the optimal shift interpolates smoothly between aligned and antialigned configurations (Fig. 5 *B* and *C* and cf. Fig. 3*B*, second row), suggesting a second-order Landau–Ginzburg phase transition (30).

### Outliers in Natural Image Statistics Modify the Transition from Aligned to Antialigned Mosaics.

The analysis above identifies output noise and image (stimulus) outliers as driving optimal encoding strategies toward higher neural response thresholds, which we claim is the key factor mediating the phase transition from aligned to antialigned mosaics. However, this analysis makes numerous simplifying assumptions, including a restriction to one dimension. Thus, we sought to test whether these predictions generalized to a less-restricted two-dimensional model in which image statistics were manipulated.

First, we identified image patches that generated the highest and the lowest firing rates in our fitted model (*SI Appendix*, Fig. S4). We identified these images using the firing rates under the two noise regimes, one that produced aligned and one that produced antialigned mosaics (Fig. 2*B* [$\sigma_{\text{in}}=0.1,\sigma_{\text{out}}=1.0$] and Fig. 2*D* [$\sigma_{\text{in}}=0.4,\sigma_{\text{out}}=3.0$]). In both noise regimes, the 100 image patches producing the highest firing rates were extreme luminance values (nearly all black or all white) or extreme contrast values (e.g., contained one or more edges between black and white regions). On the other hand, the 100 images producing the lowest firing rates were nearly homogeneous gray patches (*SI Appendix*, Fig. S4). A two-dimensional histogram of the mean and SD across the pixels of all of the available sample image patches confirmed that these images exhibited outlier mean or contrast values (*SI Appendix*, Fig. S4*E*). Thus, under both noise regimes the largest firing rates (across all neurons) were produced by rare, outlier images.

**Fig. 6.**
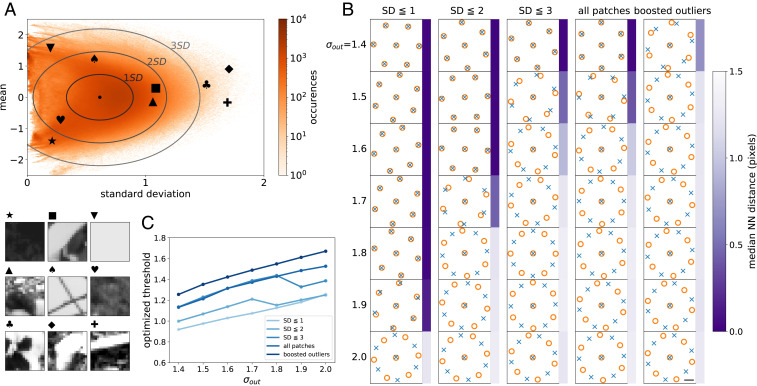
Outlier image patches drive an antialigned relationship between ON/OFF receptive field mosaics via higher optimal thresholds. (*A*) Distribution of all 18,587,848 image patches from the dataset, plotted as a two-dimensional histogram with respect to the mean and the SD of each image patch (orange). A few example patches are shown in the lower left, with the corresponding locations in the histogram marked with matching symbols. The means are centered at zero (SD = 0.575), and the SD values are centered at 0.623 (SD = 0.187). Ellipses in the histogram represent data within one to three SDs of the mean, which we use to construct artificial datasets with varying contributions of outliers. (*B*) Optimized ON and OFF receptive field mosaics using the image patches within the boundaries denoted in *A* with $\sigma_{\text{in}}$ = 0.4, $\sigma_{\text{out}}$ = 1.4 to 2.0, plotted with the same scheme as in Fig. 3. As predicted, when trained on image sets that systematically excluded outliers (i.e., had lighter-tailed distributions), phase transitions happened only at larger values of output noise. The fourth column denoted as “all patches” used the full dataset as in Fig. 3. The last column, denoted as “boosted outliers,” used an augmented dataset to which we added horizontal and vertical mirror flips of patches outside the 1.5 SD circle, thereby creating a distribution with even more outliers. This corresponds to the top 20% of z-scores among all patches in the dataset. Here, the phase transition happened at lower output noise values. The scale bar shows a one-pixel distance. (*C*) Optimal thresholds as a function of output noise, using input distributions with different levels of outliers. As predicted, the optimal thresholds tended to increase as the input distribution contained more outliers, causing the phase transition to happen at a lower output noise level.

We next tested what impact these outlier images exert on the phase transition between aligned and antialigned mosaics. We reran the “one-shape” model (e.g., Fig. 3*A*) but with the altered image sets that either reduced or increased the frequency of outlier images. From the distribution of all of the 18,587,848 possible image patches in the dataset (Fig. 6*A*) we considered a two-dimensional space composed of the z scores of the patch mean and SD values, and we drew the unit circle in this two-dimensional space. Within this space, we considered regions within one to three SDs from the mean of the average pixel intensity (per image) and from the mean of the variance over pixel intensities (per image). We then trained the one-shape model (see Fig. 3) on only the patches within each boundary (Fig. 6*B*). As predicted, we found that the transition between aligned and antialigned mosaics occurred at lower noise levels as more outlier images were included in the training set and occurred at higher noise levels when these outlier images were removed. Furthermore, these changes were mediated by an increase in activation threshold as outliers grew more prevalent (Fig. 6*C*). Thus, we were able to reproduce the effects of outlier images on the phase transition between aligned and antialigned mosaics.

## Discussion

Efficient coding theory provides a basis for understanding many features of early sensory processing. This includes the structure of individual receptive fields, how receptive fields adapt, and their mosaic-like arrangement to uniformly tile space (7, 11, 15, 21, 22). We have extended this framework to investigate how the receptive fields of distinct cell types should be spatially arranged, given sensory noise and the statistics of natural stimuli. We term this the “sensor alignment problem.” Below, we summarize the features and properties of the model that produce center-surround receptive fields, mosaics, and mosaic coordination.

### Center-Surround Receptive Fields.

The convergence of the filters to center-surround receptive fields was robust over a range of input and output noise values (*SI Appendix*, Fig. S1). However, perhaps surprisingly, convergence to center-surround filters required the higher-order correlations present in natural scenes: Training on Gaussian images with the same covariance as the natural scenes did not yield center-surround filters (*SI Appendix*, Fig. S7).

### Mosaics.

In all cases where the model produced center-surround receptive fields, it likewise produced ON and OFF cells and mosaics (see above). Moreover, when center-surround receptive fields were enforced by parameterizing filters as a difference of Gaussians, mosaics always resulted (*SI Appendix*, Figs. S2, S3, S5, and S9) even without higher-order or even long-range correlations (*SI Appendix*, Fig. S9).

### Mosaic Coordination.

The phase transition between aligned and antialigned mosaics occurred under a wide range of conditions, including natural and synthetic image sets. Gaussian-distributed images were sufficient to drive the phase transition between the aligned and antialigned states (*SI Appendix*, Fig. S5*B*). Indeed, this remained true even for images with only short-range correlations (*SI Appendix*, Fig. S9), albeit only at extremely high levels of noise.

From these relationships we can sketch the following qualitative account of the phase transition between aligned and antialigned mosaics: Given center-surround receptive fields, efficient coding argues that neurons should maximize information transmission by both distributing receptive fields to encode as much unique information as possible and reducing redundancy, which occurs when nearby cells are coactive to the same stimulus. The first intuition leads to the formation of mosaics, while the second leads, at low noise levels, to mosaic alignment. This is because optimal neuron response thresholds in the low-noise regime are lower (Fig. 4*A*), so nearby ON and OFF cells are likely to be coactive unless they are aligned, and this redundancy cost outweighs the benefits of encoding slightly different locations in the visual field (Fig. 5*D*).

However, in the high-noise or heavy-tailed neural response regimes, optimal encoding requires that individual neurons raise their response thresholds (Fig. 4*A*). This results in the decorrelation of nearby cells (31), creating an “independence zone” around each cell that grows with noise (Fig. 5 A and *E*). Receptive fields located within this distance of one another are nearly independent in their responses, allowing for nearby cells to sample distinct spatial locations without reducing encoded information through redundancy (Fig. 5*A*). The optimal configuration in this regime then becomes mosaic antialignment across a wide variety of conditions (Fig. 6 and *SI Appendix*, Fig. S9).

In previous work we have shown that real retinal mosaics of ON and OFF cells that encode similar visual features are antialigned (20). Here, we have shown that the optimal solution to the sensor alignment problem depends on both system noise and stimulus statistics. Thus, the antialignment of retinal mosaics suggests that retinal processing is optimized for low signal-to-noise conditions such as detecting dim or low-contrast stimuli (32, 33). In natural environments, the retina is probably faced with both high and low SNR conditions. When SNR is high, suboptimal processing that squanders some signal probably has little impact on sensory encoding because signal is plentiful. However, when SNR is low, the signal is precious and optimal processing is required to spare the signal from noise. This raises the question: How much does antialignment improve encoding under low SNR conditions? We previously found a ≈4% increase in mutual information for antialigned mosaics over aligned mosaics (20). While small, it is worth noting that the presence of a surround—a feature that is thought to be very important for optimal encoding of natural scenes—only improves encoding by ≈20%. Thus antialignment of mosaics is roughly 20% as important as center-surround receptive field structure.

This study connects to several other strands of work on efficient coding in the retina. As noted above, efficient coding as redundancy reduction subject to signaling costs gave theoretical weight to the idea of center-surround receptive fields as whitening filters for visual stimuli (7, 14, 15, 29, 34) that are matched to the statistics of natural scenes. However, as later work has made clear, nonlinearities, particularly response thresholds, are perhaps even more important in optimizing retinal codes (8, 31, 35). In addition, noise in both sensory transduction (inputs) and neural outputs affects coding efficiency (14, 15, 36). This work, by placing all of these factors in an optimization-based framework (21), allows for investigating the relative importance of each factor in determining the optimal spatial layout of receptive fields. Importantly, this approach relies only on information maximization arguments, and the optimization does not rely on fitting data from electrophysiological recordings, as required by recent deep-learning models of retinal coding (37, 38).

By focusing on the factors that govern the transition between aligned and antialigned mosaics from an optimal encoding standpoint, we have necessarily ignored several real biological complexities. First and foremost, the retina contains roughly 40 distinct RGC types. Our current efficient coding model only generates two types of units that encode similar visual features, but with opposite polarity, and thus we are not yet able to determine whether RGC types that encode distinct visual features should be independent or coordinated. However, a limited inspection of the mosaic relationships across RGC types that encode distinct visual features suggested that the measured receptive field mosaics were statistically independent (20). Developing an efficient coding model that is optimized to encode natural movies may yield a greater diversity of cell types (38). Many RGC types that encode particular space–time features such as direction of motion or “looming” detectors also form mosaics (39). Whether these detector grids are coordinated or should be coordinated remains an open question. Moreover, our analysis does not consider constraints on development that result in irregular mosaics, which have been shown to require local changes in receptive field shape for optimal encoding (40). Nonetheless, during the optimization process we do observe such local changes as receptive fields push against one another during mosaic formation (Movies S1 and S2). Finally, our results motivate understanding the implications of efficient coding theory to other sensory systems such as touch receptors on the skin: Might they be spatially coordinated for the efficient coding of touch (41)? More generally, these observations demonstrate that efficient coding theory can make predictions about emergent properties present in the organization of the nervous system, such as how large populations composed of multiple cell types should be arranged to optimally encode the natural environment.

## Supplementary Material

Supplementary File

Supplementary File

Supplementary File

## Data Availability

There are no data underlying this work.

## References

[1] M. Joesch, B. Schnell, S. V. Raghu, D. F. Reiff, A. Borst, On and off pathways in drosophila motion vision. Nature 468, 300–304 (2010). 21068841 10.1038/nature09545

[2] S. W. Kuffler, Discharge patterns and functional organization of mammalian retina. J. Neurophysiol. 16, 37–68 (1953). 13035466 10.1152/jn.1953.16.1.37

[3] M. Gallio, T. A. Ofstad, L. J. Macpherson, J. W. Wang, C. S. Zuker, The coding of temperature in the Drosophila brain. Cell 144, 614–624 (2011). 21335241 10.1016/j.cell.2011.01.028 PMC3336488

[4] E. C. Smith, M. S. Lewicki, Efficient auditory coding. Nature 439, 978–982 (2006). 16495999 10.1038/nature04485

[5] S. H. Chalasani., Dissecting a circuit for olfactory behaviour in caenorhabditis elegans. Nature 450, 63–70 (2007). 17972877 10.1038/nature06292

[6] C. C. Bell, Mormyromast electroreceptor organs and their afferent fibers in mormyrid fish. III. Physiological differences between two morphological types of fibers. J. Neurophysiol. 63, 319–332 (1990). 2313348 10.1152/jn.1990.63.2.319

[7] H. B. Barlow, Possible principles underlying the transformation of sensory messages. Sensory Commun. 1, 217–234 (1961).

[8] J. Gjorgjieva, H. Sompolinsky, M. Meister, Benefits of pathway splitting in sensory coding. J. Neurosci. 34, 12127–12144 (2014). 25186757 10.1523/JNEUROSCI.1032-14.2014 PMC4152610

[9] S. H. Devries, D. A. Baylor, Mosaic arrangement of ganglion cell receptive fields in rabbit retina. J. Neurophysiol. 78, 2048–2060 (1997). 9325372 10.1152/jn.1997.78.4.2048

[10] B. G. Borghuis, C. P. Ratliff, R. G. Smith, P. Sterling, V. Balasubramanian, Design of a neuronal array. J. Neurosci. 28, 3178–3189 (2008). 18354021 10.1523/JNEUROSCI.5259-07.2008 PMC2646167

[11] E. Doi., Efficient coding of spatial information in the primate retina. J. Neurosci. 32, 16256–16264 (2012). 23152609 10.1523/JNEUROSCI.4036-12.2012 PMC3537829

[12] J. L. Gauthier., Receptive fields in primate retina are coordinated to sample visual space more uniformly. PLoS Biol. 7, e1000063 (2009). 19355787 10.1371/journal.pbio.1000063 PMC2672597

[13] J. L. Gauthier., Uniform signal redundancy of parasol and midget ganglion cells in primate retina.J. Neurosci. 29, 4675–4680 (2009). 19357292 10.1523/JNEUROSCI.5294-08.2009 PMC3202971

[14] J. J. Atick, A. N. Redlich, Towards a theory of early visual processing. Neural Comput. 2, 308–320 (1990).

[15] J. J. Atick, A. N. Redlich, What does the retina know about natural scenes? Neural Comput. 4, 196–210 (1992).

[16] H. Wässle, L. Peichl, B. B. Boycott, Morphology and topography of on- and off-alpha cells in the cat retina. Proc. R. Soc. Lond. B Biol. Sci. 212, 157–175 (1981). 6166012 10.1098/rspb.1981.0032

[17] R. L. Rockhill, T. Euler, R. H. Masland, Spatial order within but not between types of retinal neurons. Proc. Natl. Acad. Sci. U.S.A. 97, 2303–2307 (2000). 10688875 10.1073/pnas.030413497 PMC15796

[18] S. J. Eglen, “Cellular spacing: Analysis and modelling of retinal mosaics” in Computational Systems Neurobiology, N. Le Novère, Ed. (Springer, 2012), pp. 365–385.

[19] J. Jang, S. B. Paik, Interlayer repulsion of retinal ganglion cell mosaics regulates spatial organization of functional maps in the visual cortex. J. Neurosci. 37, 12141–12152 (2017). 29114075 10.1523/JNEUROSCI.1873-17.2017 PMC6596818

[20] S. Roy, N. Y. Jun, E. L. Davis, J. Pearson, G. D. Field, Inter-mosaic coordination of retinal receptive fields. Nature 592, 409–413 (2021). 33692544 10.1038/s41586-021-03317-5 PMC8049984

[21] Y. Karklin, E. P. Simoncelli, “Efficient coding of natural images with a population of noisy linear-nonlinear neurons” in Advances in Neural Information Processing Systems, M. I. Jordan, Y. LeCun, S. A. Solla, Eds. (MIT Press, 2011), pp. 999–1007. PMC4532291 26273180

[22] N. Ratliff, M. Zucker, J. A. Bagnell, S. Srinivasa., “Chomp: Gradient optimization techniques for efficient motion planning” in 2009 IEEE International Conference on Robotics and Automation (IEEE, 2009), pp. 489–494.

[23] E. Doi, M. S. Lewicki, “A theory of retinal population coding” in Advances in Neural Information Processing Systems, J. Platt, D. Koller, Y. Singer, S. Roweis, Eds. (MIT Press, 2007), pp. 353–360.

[24] D. P. Kingma, J. Ba, “Adam: {A} Method for stochastic optimization” in 3rd International Conference on Learning Representations, Y. Bengio, Y. LeCun, Eds. (ICLR, San Diego, CA, 2015).

[25] M. Meister, M. J. Berry 2nd, The neural code of the retina. Neuron 22, 435–450 (1999). 10197525 10.1016/s0896-6273(00)80700-x

[26] E. J. Chichilnisky, A simple white noise analysis of neuronal light responses. Network 12, 199–213 (2001). 11405422

[27] S. A. Baccus, M. Meister, Fast and slow contrast adaptation in retinal circuitry. Neuron 36, 909–919 (2002). 12467594 10.1016/s0896-6273(02)01050-4

[28] D. L. Ruderman, The statistics of natural images. Network 5, 517–548 (1994).

[29] S. B. Laughlin, Energy as a constraint on the coding and processing of sensory information. Curr. Opin. Neurobiol. 11, 475–480 (2001). 11502395 10.1016/s0959-4388(00)00237-3

[30] J. J. Binney, N. J. Dowrick, A. J. Fisher, M. E. Newman, The Theory of Critical Phenomena: An Introduction to the Renormalization Group (Oxford University Press, 1992).

[31] X. Pitkow, M. Meister, Decorrelation and efficient coding by retinal ganglion cells. Nat. Neurosci. 15, 628–635 (2012). 22406548 10.1038/nn.3064 PMC3725273

[32] N. K. Dhingra, Y. H. Kao, P. Sterling, R. G. Smith, Contrast threshold of a brisk-transient ganglion cell in vitro. J. Neurophysiol. 89, 2360–2369 (2003). 12611985 10.1152/jn.01042.2002

[33] G. D. Field, A. P. Sampath, Behavioural and physiological limits to vision in mammals. Philos. Trans. R. Soc. Lond. B Biol. Sci. 372, 20160072 (2017). 28193817 10.1098/rstb.2016.0072 PMC5312022

[34] M. V. Srinivasan, S. B. Laughlin, A. Dubs, Predictive coding: A fresh view of inhibition in the retina. Proc. R. Soc. Lond. B Biol. Sci. 216, 427–459 (1982). 6129637 10.1098/rspb.1982.0085

[35] J. Gjorgjieva, M. Meister, H. Sompolinsky, Functional diversity among sensory neurons from efficient coding principles. PLOS Comput. Biol. 15, e1007476 (2019). 31725714 10.1371/journal.pcbi.1007476 PMC6890262

[36] K. Röth, S. Shao, J. Gjorgjieva, Efficient population coding depends on stimulus convergence and source of noise. PLoS Comput. Biol. 17, e1008897 (2021). 33901195 10.1371/journal.pcbi.1008897 PMC8075262

[37] N. Maheswaranathan, D. B. Kastner, S. A. Baccus, S. Ganguli, Inferring hidden structure in multilayered neural circuits. PLOS Comput. Biol. 14, e1006291 (2018). 30138312 10.1371/journal.pcbi.1006291 PMC6124781

[38] S. Ocko, J. Lindsey, S. Ganguli, S. Deny, “The emergence of multiple retinal cell types through efficient coding of natural movies” in Advances in Neural Information Processing Systems, S. Bengio et al., Eds. (Curran Associates, 2018), pp. 9389–9400.

[39] D. I. Vaney, B. Sivyer, W. R. Taylor, Direction selectivity in the retina: Symmetry and asymmetry in structure and function. Nat. Rev. Neurosci. 13, 194–208 (2012). 22314444 10.1038/nrn3165

[40] Y. S. Liu, C. F. Stevens, T. O. Sharpee, Predictable irregularities in retinal receptive fields. Proc. Natl. Acad. Sci. U.S.A. 106, 16499–16504 (2009). 19805327 10.1073/pnas.0908926106 PMC2741481

[41] E. D. Kuehn, S. Meltzer, V. E. Abraira, C. Y. Ho, D. D. Ginty, Tiling and somatotopic alignment of mammalian low-threshold mechanoreceptors. Proc. Natl. Acad. Sci. U.S.A. 116, 9168–9177 (2019). 30996124 10.1073/pnas.1901378116 PMC6511030

