# Supplementary material for: Scene statistics and noise determine the relative arrangement of receptive field mosaics
Source: Proc Natl Acad Sci U S A. 2021 Sep 23;118(39):e2105115118. doi: 10.1073/pnas.2105115118 (PMC8488585; doi:10.1073/pnas.2105115118)
Supplement: Supplementary File [file pnas.2105115118.sapp.pdf]

PNAS
www.pnas.org

Supplementary Information for

Scene statistics and noise determine the relative arrangement of receptive field mosaics

Na Young Jun, Greg D. Field and John Pearson

John Pearson.
E-mail: john.pearson@duke.edu

This PDF file includes:

Supplementary text
Figs. S1 to S12 (not allowed for Brief Reports)
Legends for Movies S1 to S2
SI References

Other supplementary materials for this manuscript include the following:

Movies S1 to S2

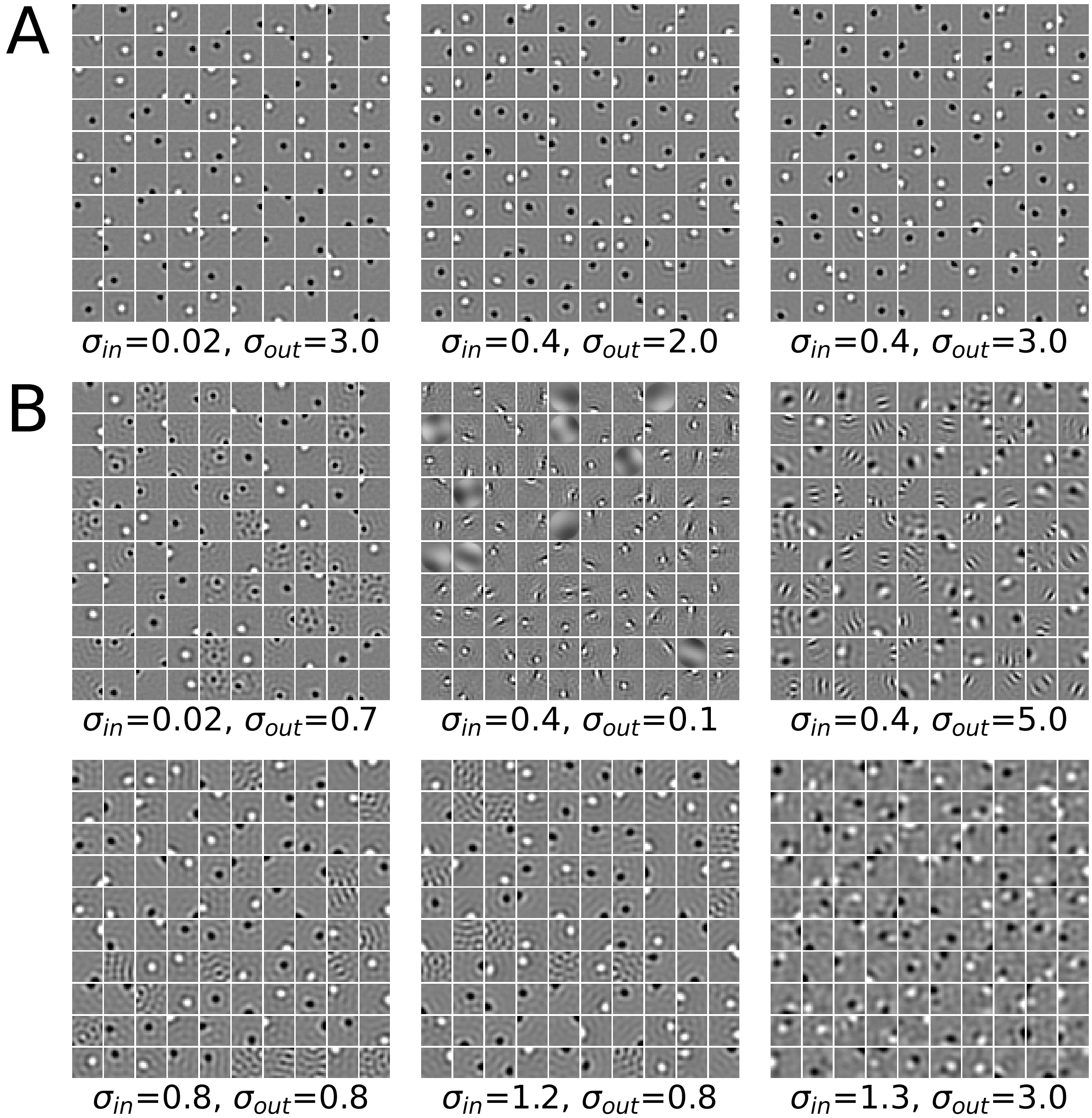

Figure S1. Circular ON and OFF receptive fields are robustly obtained within a range of input and output noise.
A. With input noise in the range between 0.00 to 0.75 and output noise between 0.75 to 3.00, circular center-surround ON and OFF receptive fields are obtained reliably. **B.** Outside these ranges, i.e., when input noise is higher than 0.75 or output noise is lower than 0.75, either Gabor-shaped or noisy kernels are obtained.

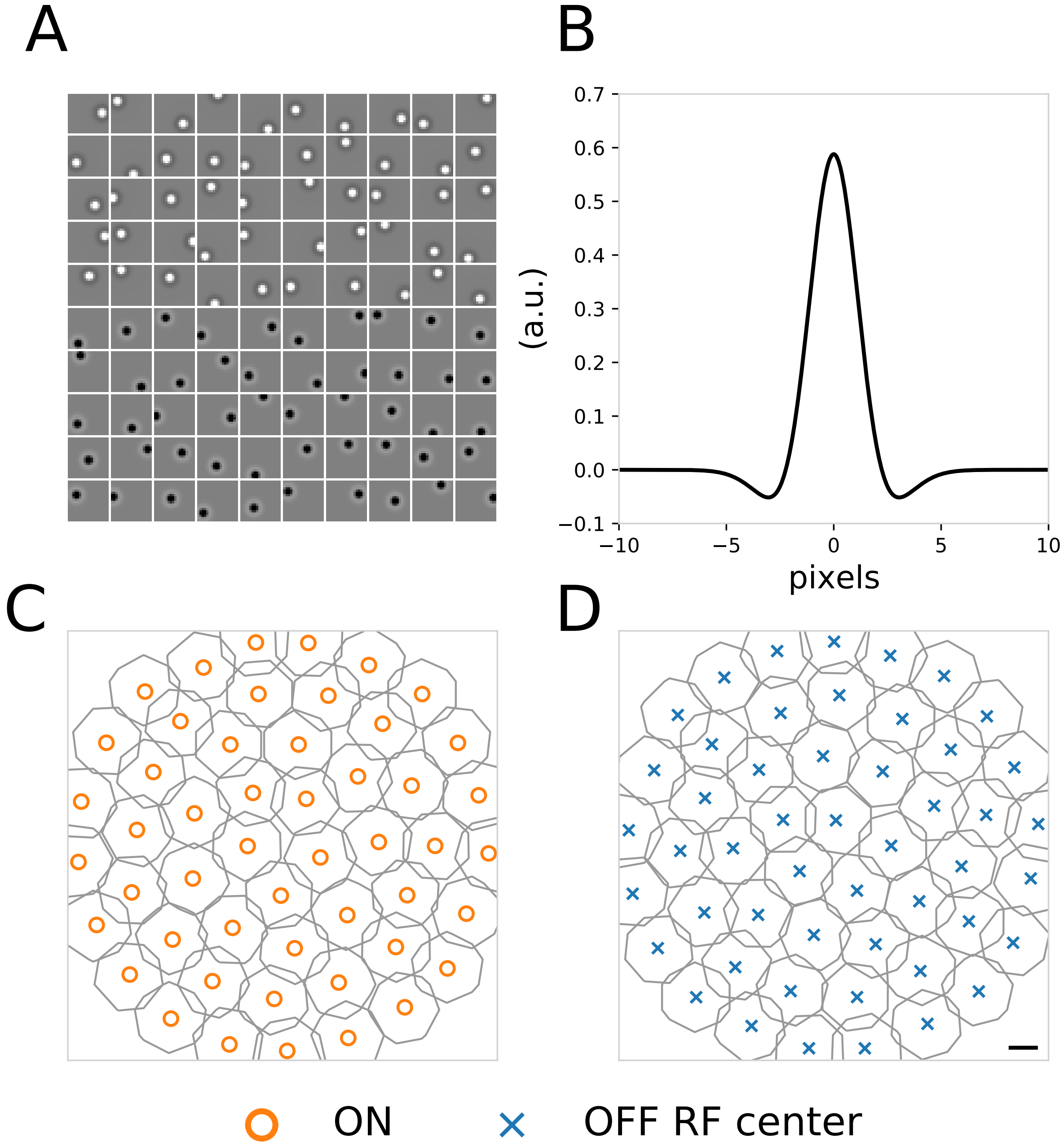

Figure S2. When all filters share the same kernel, filters form mosaics.
A. A plot of 100 kernels that maximize information when all kernels are constrained to take the same shape but are free to move their centers (cf. **Fig. 1C**) **B.** The radial shape function is parameterized as a difference of Gaussian curves. Learned radial shape parameters are: a=0.3332, b=0.1559, c=0.4121. **C.** Learned ON kernels sharing the same radial shape fill the field (similar to **Fig. 1D**). **D.** Learned OFF kernels sharing the same radial shape fill the field (similar to **Fig. 1E**). Scale bar is width of one image pixel.

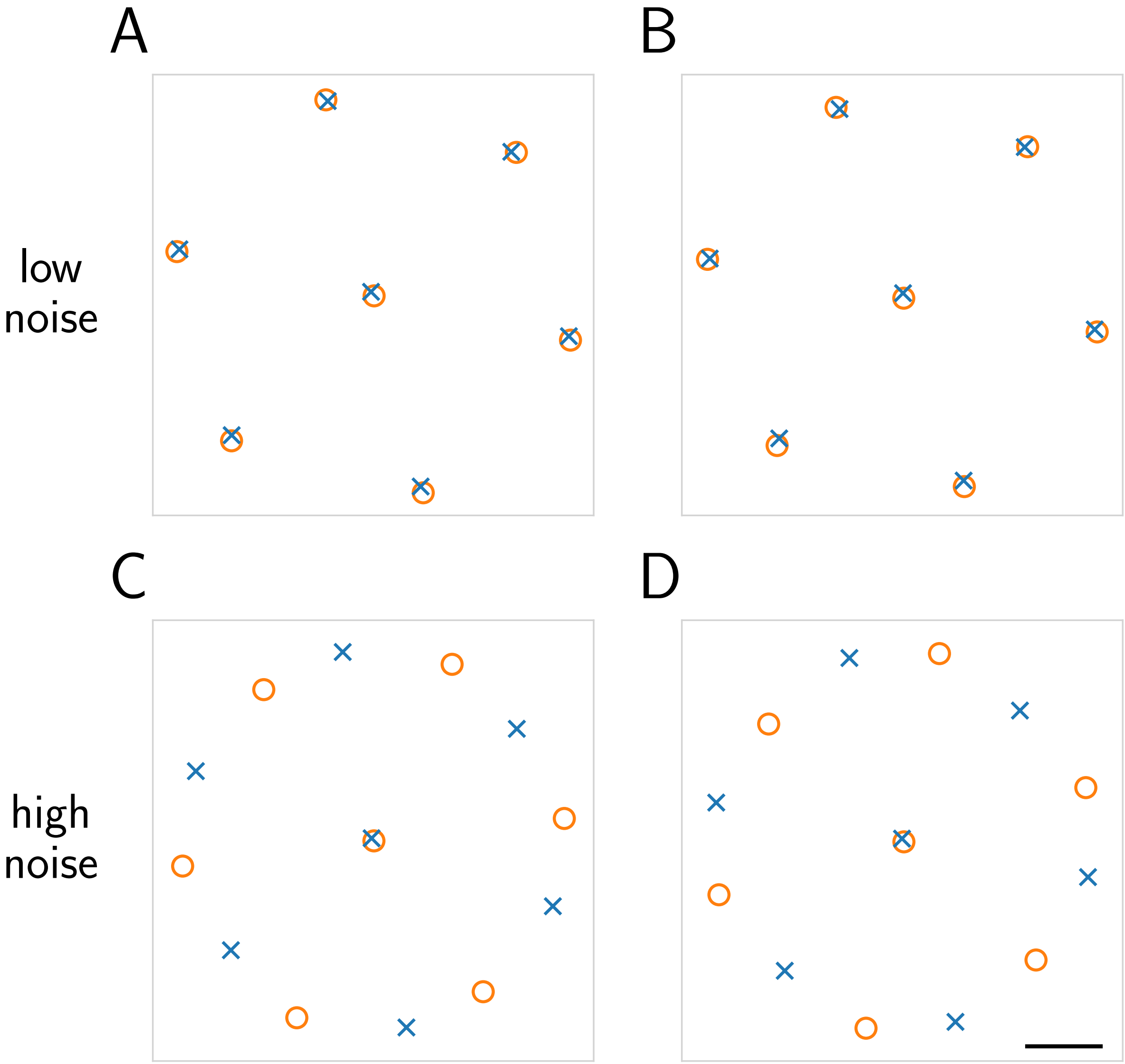

Figure S3. A one-shape model captures the dependence of mosaic arrangements on input and output noise.
Receptive field centers for ON (orange circle) and OFF (blue x) cells under different sets of noise parameters. **A:** (σ_{in}=0.02, σ_{out}=1.0), **B:** (σ_{in}=0.1, σ_{out}=1.0), **C:** (σ_{in}=0.4, σ_{out}=2.0), **D:** (σ_{in}=0.4, σ_{out}=3.0). The first two parameter sets result in aligned mosaics, while the latter two, at higher levels of output noise, are anti-aligned. Scale bar is width of one image pixel.

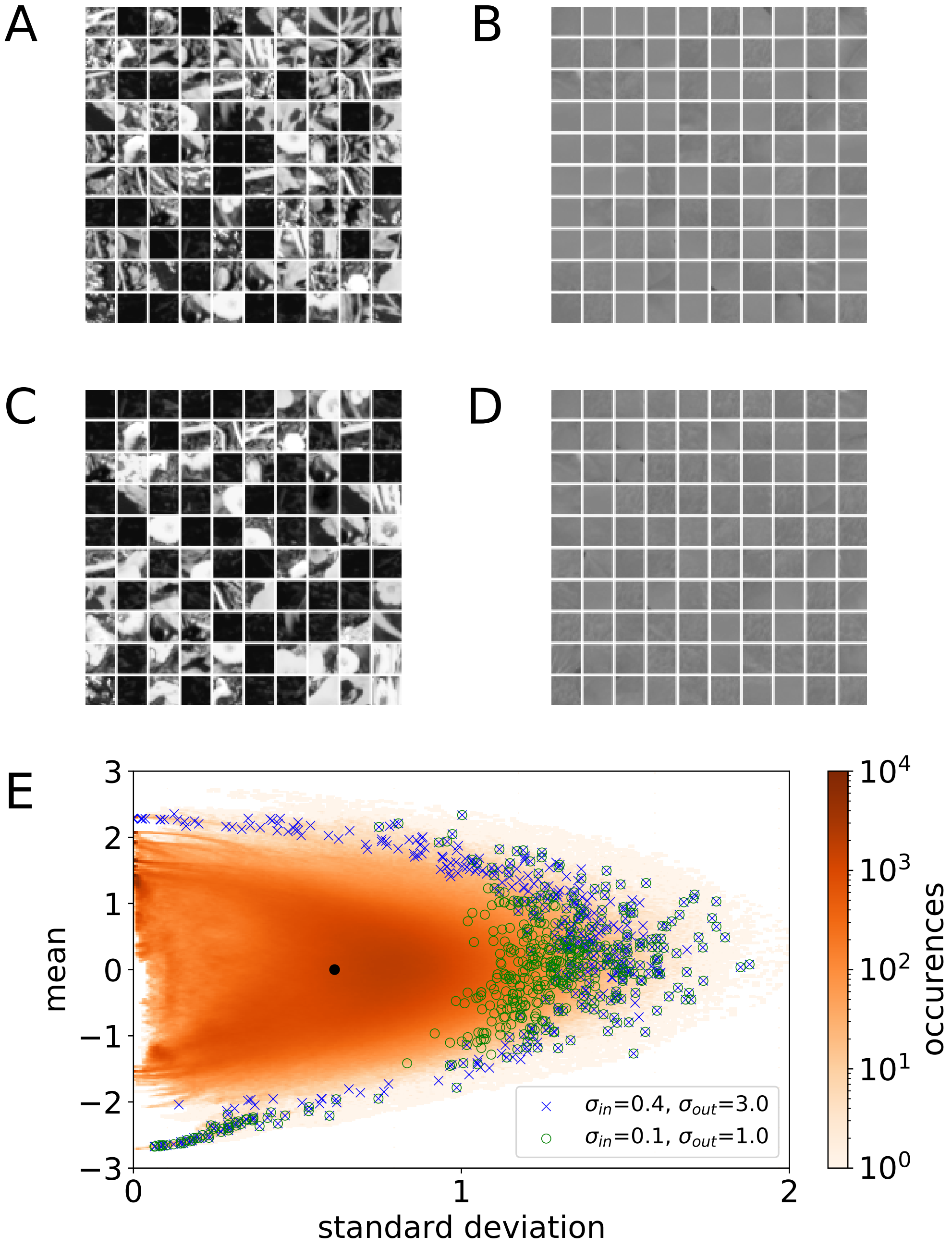

Figure S4. Image patches with the highest firing rates and lowest firing rates (among 100,000 randomly sampled image patches) are distinct in both the high noise (σ_{in}=0.4, σ_{out}=3.0) and the low noise (σ_{in}=0.1, σ_{out}=1.0) regimes.
Most of the image patches with the highest firing rates are outliers of the overall image patch distribution, containing high contrast edges or particularly high or low mean intensity. This is also evident in firing rate outputs: Using the kernels optimized under the two noise regimes, as shown in **Fig. 2B** and **Fig. 2D**, we calculated the firing rates of 100,000 randomly sampled image patches and overlaid the top 100 patches with the highest firing rates over the z-score histogram in **Fig. 6A**. **A.** 100 image patches with the highest firing rates under σ_{in}=0.1, σ_{out}=1.0. Images are either extremely dark or contain distinct edges, including those with more complicated edges compared to **C**, and had firing rates of 3.07±0.23. **B.** 100 image patches with the lowest firing rates under σ_{in}=0.1, σ_{out}=1.0. The 100 images with the lowest firing rates were also visually similar and had firing rates of 0.161±0.003. **C.** 100 image patches with the highest firing rates under σ_{in}=0.4, σ_{out}=3.0. Images are either dark or contain distinct edges, with firing rates of 4.39±0.54. **D.** 100 image patches with the lowest firing rates under σ_{in}=0.4, σ_{out}=3.0. Patches were almost entirely gray, with firing rates of 0.036±0.001. **E.** A 2-D histogram showing the mean and standard deviation of all of the available sample image patches (in orange, occurrences in log scale, same as **Fig. 6A**) with the image patches in **A** (blue x) and the image patches in **B** (green o). In the low-noise condition, the outliers are a greater portion of the tail (green o).

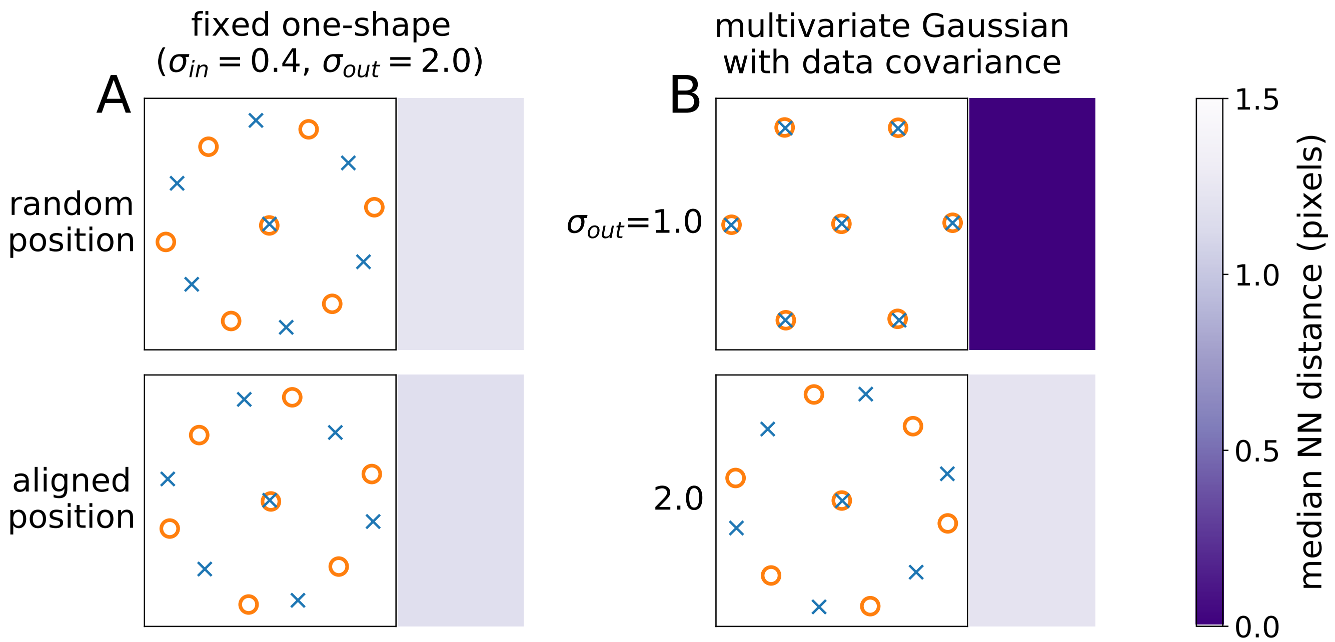

Figure S5. The transition between aligned and anti-aligned occurs when kernel shape is fixed and with Gaussian images.
A. When kernel parameters are not learnable (fixed to a, b, c = [0.258, 0.162, 0.648]) and noise is high, the optimal configuration remains anti-alignment. This occurs when the receptive fields are initialized to random positions (top) or aligned (bottom) positions (cf. **Fig. 5B** top row of "all patches"). Here σ_{in}=0.4, σ_{out}=1.0. **B.** The transition from aligned to anti-aligned mosaics occurs when images are drawn from a covariance-matched Gaussian distribution. This matches the assumptions of the analytical model in Supplementary Text 1 and demonstrates that heaver-than-Gaussian tails are not necessary for anti-alignment to be the favored state.

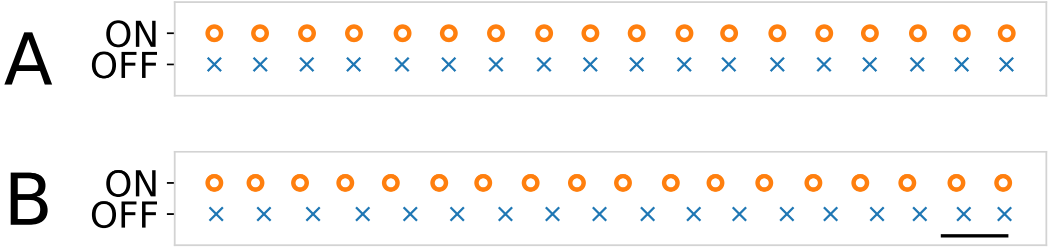

Figure S6. The phase transition occurs in a 1-dimensional model. Optimal receptive field centers of 18 ON and 18 OFF receptive fields in a 1-by-64 pixel input. Receptive field centers for ON (orange circle) and OFF (blue x) cells are optimized under two sets of noise parameters, A: (σ_{in}=0.4, σ_{out}=1.0), B: (σ_{in}=0.4, σ_{out}=3.0). Noise parameters in **A** produce aligned mosaics, while those in **B** produce anti-aligned mosaics. Scale bar is width of one image pixel.

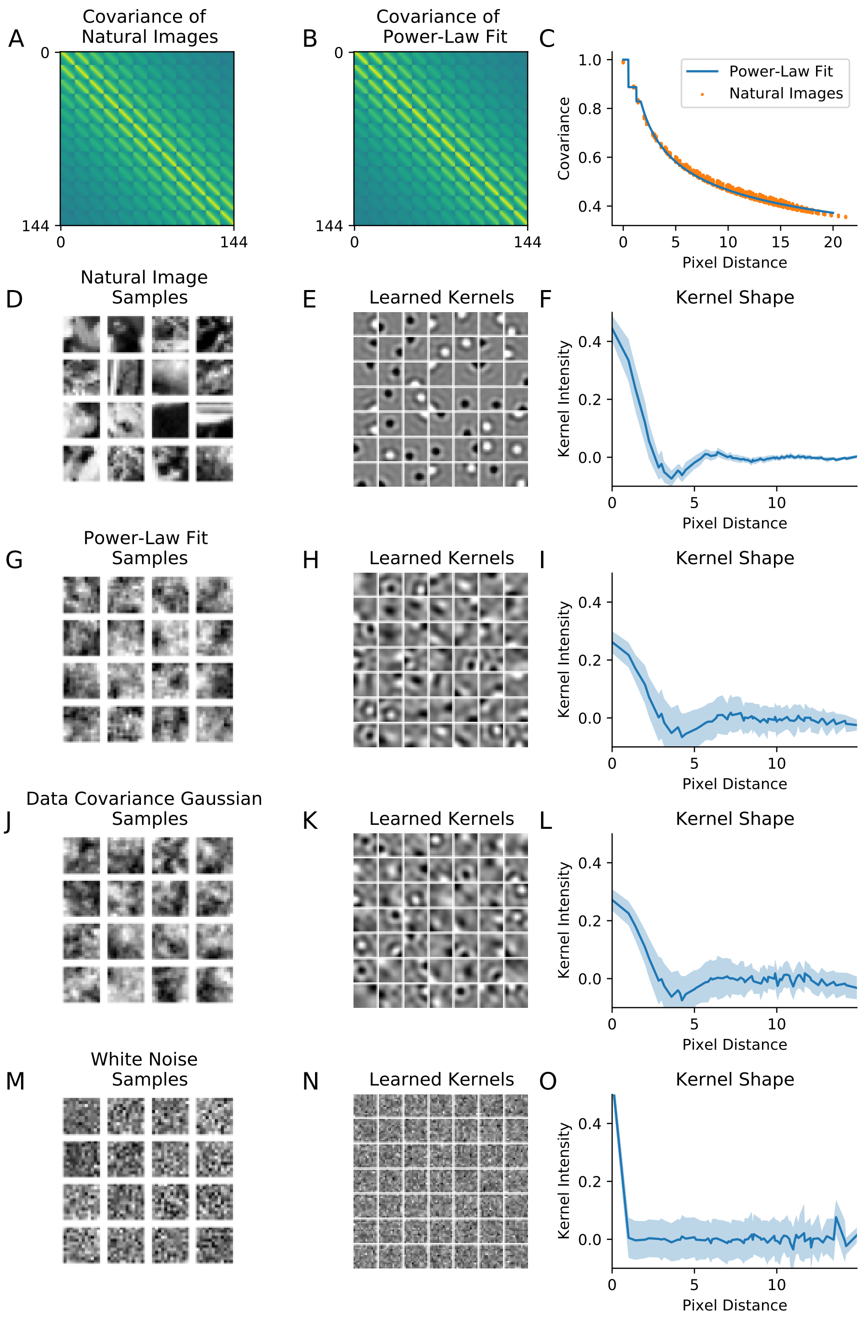

Figure S7. Learned kernels do not converge to circular center-surround shapes for non-natural image distributions. A. Covariance matrix of the natural images, calculated from 100,000 random image patches from (1). **B.** Covariance matrix constructed from the power-law fit of the values in **A**. **C.** Covariance of the 100,000 natural image patches (orange dots) and the power-law fit of the covariance (blue curve) defined as: $C_{ijkl} = \min(1, 0.9765r^{-0.322})$ where $r = \sqrt{(i-k)^2 + (j-l)^2}$. **D.** Natural image samples. **E.** Learned kernels when natural image samples such as **D** are the inputs to the model. **F.** Mean (blue curve) and standard deviation (light blue shade) of the kernel intensities as a function of the pixel distance from the location of the peak intensity (regardless of the ON and OFF centers). **G.** Image samples drawn from a multivariate Gaussian distribution with zero mean and the power-law fit covariance in **B**. **H.** Learned kernels when samples as in **G** are used as inputs. **I.** Same as **F** but with the kernels in **H**. **J.** Image samples drawn from a multivariate Gaussian with zero mean and the empirical covariance of natural images in **A**. **K.** Learned kernels when samples from **J** are used as inputs. **L.** Same as **F** but with the kernels in **K**. **M.** White noise image samples where each pixel is drawn i.i.d. from the standard normal distribution. **N** Learned kernels when the white noise samples such as those in **M** are the inputs of the model. **O.** Same as **F** but with the kernels in **N**. Only in the case of natural images do center-surround kernels emerge from training.

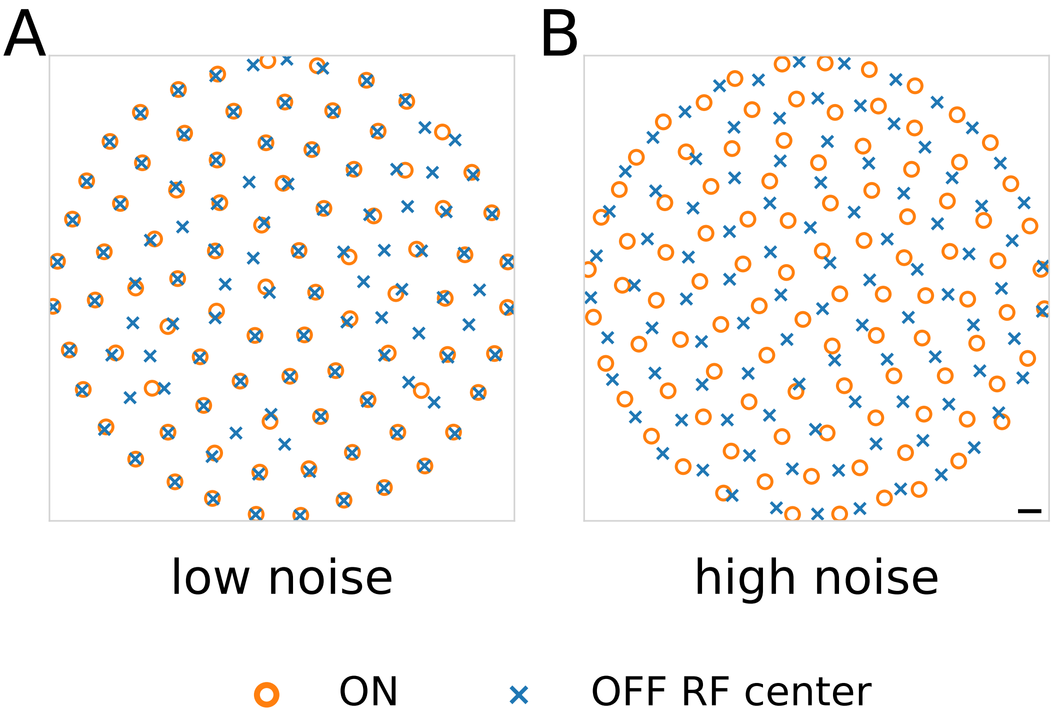

Figure S8. Larger image patches also produce anti-aligned mosaics. Receptive field centers for ON (orange circles) and OFF (blue Xs) cells under different noise configurations for patches of 25 x 25 pixels and 196 units. **A**: (σ_{in}=0.4, σ_{out}=1.0), **B**: (σ_{in}=0.4, σ_{out}=3.0). Noise parameters in **A** produce aligned mosaics, while those in **B** produce anti-aligned mosaics. Scale bar is width of one image pixel.

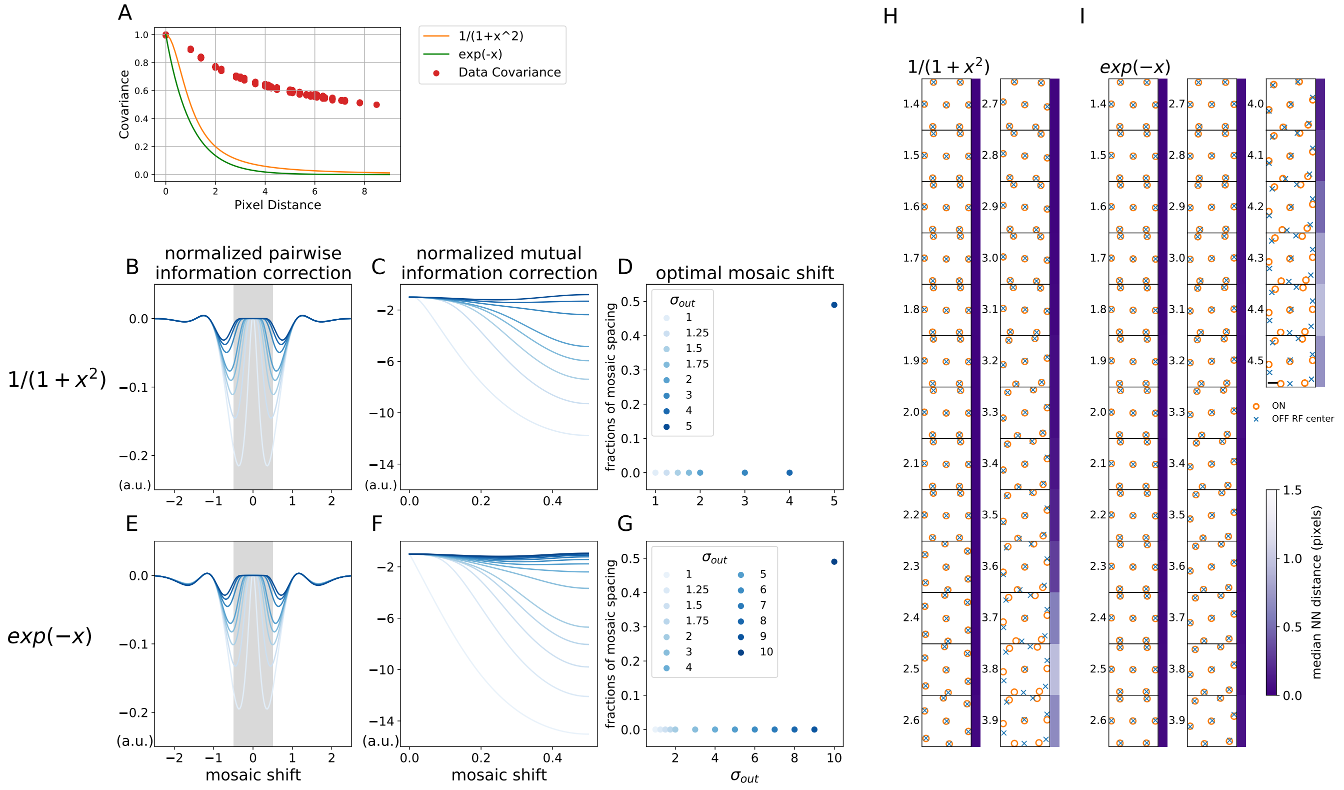

Figure S9. The transition to anti-aligned mosaics occurs with short-range correlations. A. Covariance of the 100,000 natural image patches (red dots) with long-range correlations, and two alternative correlation structures: $1/(1+x^2)$ (orange curve) and $\exp(-x)$ (green curve). **B-D.** Replication of **Fig. 5A-C** when a model with assumed center-surround kernels is trained on images drawn from a multivariate Gaussian distribution with zero mean and the covariance function $1/(1+x^2)$. Unlike with natural images, anti-alignment occurs at very high levels of output noise. **E-G.** Same as **B-D** but when trained with the image samples drawn from a multivariate Gaussian distribution with zero mean and the $\exp(-x)$ covariance. **H.** Optimized ON and OFF receptive field mosaics of the 2D one-shape model using image patches with the $1/(1+x^2)$ covariance. The phase transition is observed around σ_{out}=3.7, which is a higher noise than that producing the transition for natural images in **Fig. 3**. **I.** Same as **H** but for $\exp(-x)$ covariance. The phase transition is observed around σ_{out}=4.3, which is even higher than the case in **H**.

Movie S1. Evolution of receptive fields over training. Top. Example receptive fields for an OFF (left) and ON (right) receptive field over the course of model training. Both are initialized as white noise filters and quickly develop center-surround shapes when trained on natural image patches. Bottom. Model nonlinearities for the same two cells. Here, both thresholds and gains increase during training.

Movie S2. Evolution of mosaics over training. Example mosaics for ON (left) and OFF (right) cells over the course of model training. Plotted contours are the same conventions as Fig. 1. Because the model optimizes encoded information, redundancy in the form of overlapping receptive fields of the same polarity is penalized, resulting in a "repulsive" interaction between neighboring receptive fields.

Supporting Information Text

1. Modeling details

A. Mutual information in the linear-nonlinear model.

We follow the conventions of (2). Let $\mathbf{x} \in \mathbb{R}^D$ be a vectorized representation of an input image. We assume J neurons characterized by linear filters $\mathbf{w}_j$ ($\|\mathbf{w}_j\| = 1$) for $j = 1 \dots N$, which form the columns of a matrix $\mathbf{W} \in \mathbb{R}^{D \times J}$. We assume pixelwise input noise to each image $\mathbf{n}_x \sim \mathcal{N}(0, \sigma^2_{\text{in}})$ and output noise in firing rates $\mathbf{n}_r \sim \mathcal{N}(0, \sigma^2_{\text{out}})$ so that the random input image and firing rate are $\mathbf{X} = \mathbf{x} + \mathbf{n}_x$ and $\mathbf{R} = \mathbf{r} + \mathbf{n}_r$, respectively. With these conventions, the firing rate for the jth neuron given input $\mathbf{x}$ is

$$r_j = \gamma_j \eta(\mathbf{w}_j^\top (\mathbf{x} + \mathbf{n}_x) - \theta_j) + n_{r,j}, \quad [1]$$

with γ_j the neuron gain, θ_j its response threshold, and η the softplus nonlinearity described in Methods. Note that, in principle, this firing can be negative for sufficiently large and negative $n_{r,j}$. As in (2), we notheless treat **Eq 1** as a useful approximation.

Under these assumptions, the covariance matrix of firing rates given input image is

$$\mathbf{C}_{r|x} = \mathbf{G}\mathbf{W}^\top \mathbf{C}_{\text{in}} \mathbf{W}\mathbf{G} + \mathbf{C}_{\text{out}} \quad [2]$$

with $\mathbf{C}_{\text{in}} = \sigma^2_{\text{in}} \mathbb{1}$, $\mathbf{C}_{\text{out}} = \sigma^2_{\text{out}} \mathbb{1}$, and $\mathbf{G}(\mathbf{x}) = \text{diag}(\gamma_j \eta(\mathbf{w}^\top(\mathbf{x} + \mathbf{n}_x) - \theta))$ a matrix of derivatives of the nonlinearities. As in (2), we make a local Gaussian approximation to the image distribution with covariance $\mathbf{C}_x$, allowing us write the posterior $p(\mathbf{x}|\mathbf{r})$ as Gaussian with covariance

$$\begin{aligned} \mathbf{C}_{x|r} &= \left(\mathbf{C}_x^{-1} + \mathbf{W}\mathbf{G}\mathbf{C}_{r|x}^{-1}\mathbf{G}\mathbf{W}^\top\right)^{-1} \\ &= \left(\mathbf{C}_x^{-1} + \mathbf{W}\mathbf{G}\left(\mathbf{G}\mathbf{W}^\top \mathbf{C}_{\text{in}} \mathbf{W}\mathbf{G} + \mathbf{C}_{\text{out}}\right)^{-1} \mathbf{G}\mathbf{W}^\top\right)^{-1} \end{aligned} \quad [3]$$

and the conditional (differential) entropy as

$$H(\mathbf{X}|\mathbf{R}) = \mathbb{E}_{\mathbf{x}}\left[\frac{1}{2}\log\det\left(2\pi e \mathbf{C}_{x|r}\right)\right]. \quad [4]$$

Since $H(\mathbf{X})$ is an unknown constant, maximizing the mutual information $\mathcal{I}(\mathbf{X};\mathbf{R}) = H(\mathbf{X}) - H(\mathbf{X}|\mathbf{R})$ involves *minimizing* **Eq 4** subject to a cost per spike that sets constrains the mean firing rate of each neuron:

$$\begin{aligned} &\underset{\mathbf{W},\gamma,\boldsymbol{\theta}}{\text{maximize}} \quad \mathbb{E}_{\mathbf{x}}\left[\log \frac{\det\left(\mathbf{G}\mathbf{W}^\top(\mathbf{C}_x + \mathbf{C}_{\text{in}})\mathbf{W}\mathbf{G} + \mathbf{C}_{\text{out}}\right)}{\det\left(\mathbf{G}\mathbf{W}^\top \mathbf{C}_{\text{in}}\mathbf{W}\mathbf{G} + \mathbf{C}_{\text{out}}\right)}\right] \\ &\text{subject to} \quad \mathbb{E}\left[\mathbf{R}\right] = 1, \end{aligned} \quad [5]$$

where the optimization objective follows from **Eqs 3** and **4** via the Matrix Inversion Lemma and we implement the constraint using an augmented Lagrangian method as detailed in Methods. Finally, the following rewriting of **Eq 5** will be useful, particularly in our analysis of the single-neuron case:

$$\begin{aligned} &\frac{\det\left(\mathbf{G}\mathbf{W}^\top(\mathbf{C}_x + \mathbf{C}_{\text{in}})\mathbf{W}\mathbf{G} + \mathbf{C}_{\text{out}}\right)}{\det\left(\mathbf{G}\mathbf{W}^\top \mathbf{C}_{\text{in}}\mathbf{W}\mathbf{G} + \mathbf{C}_{\text{out}}\right)} \\ &= \det\left(\mathbb{I} + \mathbf{G}\mathbf{W}^\top \mathbf{C}_x \mathbf{W}\mathbf{G}\left(\mathbf{G}\mathbf{W}^\top \mathbf{C}_{\text{in}}\mathbf{W}\mathbf{G} + \mathbf{C}_{\text{out}}\right)^{-1}\right). \end{aligned} \quad [6]$$

B. Analytic model of alignment in 1-D.

To better understand the optimization problem posed in **Eq 5**, particularly the contributions of kernel shape, image statistics, noise, and response thresholds to the transition from aligned to anti-aligned mosaics, we consider a simplified model that nonetheless exhibits the same behavior. In particular:

1. We restrict ourselves to a one-dimensional space in which ON and OFF mosaics are assumed to be an equidistant array of receptive field centers (distance = 1) for both mosaics. The ON cells are located at positions $z_j = j$, $j \in \mathbb{Z}$, and the mosaics are allowed to rigidly shift relative to each other, so that OFF cells are located at $\bar{z}_j = j + \phi$, $\phi \in [0, 0.5]$.

2. We assume a fixed kernel shape for all neurons, meaning that receptive fields differ only in their center locations: $w_j(z) = \pm\kappa(|z - z_j|)$ for a kernel shape function $\kappa(z)$, where z_j is the location of j-th neuron's kernel center.

3. We likewise assume identical nonlinearities for all units: $f_j(y) = \gamma(y - \theta)_+$, where γ is the gain, θ the response threshold parameter, and $(\cdot)_+ = \max(\cdot, 0)$ denotes positive rectification.

4. Images $\mathbf{x}$ are assumed to follow a multivariate Gaussian distribution $\mathcal{N}(0, \mathbf{C}_x)$, where the covariance between locations z_i and z_j only depends on $|z_i - z_j|$, defined as a function $C_x(z)$. In analogy with the finding that the amplitudes of natural images decrease as $\sim 1/k$ with k the spatial frequency (power spectral density $\sim 1/k^2$), for our numerical examples, we likewise assume a *one-dimensional* scale-free distribution $\sim 1/\sqrt{k}$ (power spectrum $\sim 1/k$). As shown in **Fig. S5B**, the phase transition still takes place even with this Gaussian image assumption.

Now, for a pair of neurons j and k, the scalars $\mathbf{w}_j^\top \mathbf{w}_k$ and $\mathbf{w}_j^\top \mathbf{C}_x \mathbf{w}_k$ depend only on $|z_j - z_k|$, the distance between their centers, which we write in the frequency domain as:

$$\begin{aligned} \mathbf{w}_j^\top \mathbf{w}_k &= \frac{1}{2\pi}\int e^{i(z_j - z_k)\omega}|\kappa(\omega)|^2 d\omega \\ &\stackrel{\text{def}}{=} f(|z_j - z_k|) \end{aligned} \quad [7]$$

and

$$\begin{aligned} \mathbf{w}_j^\top \mathbf{C}_x \mathbf{w}_k &= \frac{1}{2\pi}\int e^{i(z_j - z_k)\omega} C_x(\omega)|\kappa(\omega)|^2 d\omega \\ &\stackrel{\text{def}}{=} g(|z_j - z_k|), \end{aligned} \quad [8]$$

where $C_x(\omega)$ and $\kappa(\omega)$ are Fourier transforms of $C_x(z)$ and $\kappa(z)$, respectively. Using any $\mathbf{w}$, we can define the constants f_0 and g_0 for the special case where $z_j = z_k$:

$$f_0 \stackrel{\text{def}}{=} f(0) = \mathbf{w}^\top \mathbf{w} = \|\mathbf{w}\| = 1 \quad [9]$$

$$g_0 \stackrel{\text{def}}{=} g(0) = \mathbf{w}^\top \mathbf{C}_x \mathbf{w} \quad [10]$$

where we used our assumption that the kernel $\mathbf{w}$ is a unit vector in **Eq 9**. An example of these functions is shown in **Fig. S10**.

The single-neuron case: $\mathcal{I}_1$.

We first consider the mutual information capacity of a single neuron in isolation. Since we have assumed all units to be identical, the same expression holds for all ON and OFF cells, and we let

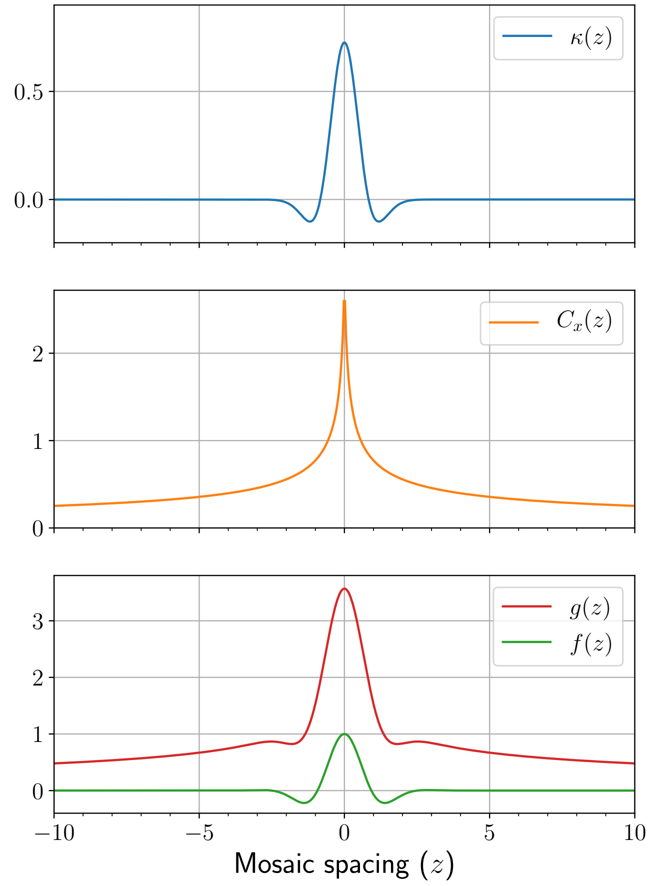

Figure S10. Plots of $\kappa(z)$, $C_x(z)$, $f(z)$, and $g(z)$ using trained parameters from a one-shape model and an approximation of the data covariance: $\kappa(z) \propto e^{-1.76z^2} - 0.65e^{-1.12z^2}$ and $C_x(z) = \frac{0.8}{\sqrt{|z|+0.53}}$.

$\mathcal{I}_1 \stackrel{\text{def}}{=} -H(X|R)$ in this single-channel case. From **Eq 6**, this quantity is

$$\begin{aligned}\mathcal{I}_1 &= p_1 \log\left(1 + \frac{\gamma^2 \mathbf{w}^\mathsf{T}\mathbf{C}_x\mathbf{w}}{\gamma^2\sigma_{\text{in}}^2 + \sigma_{\text{out}}^2}\right) \\ &= p_1 \log\left(1 + \frac{g_0}{\sigma_{\text{in}}^2 + \sigma_{\text{out}}^2\gamma^{-2}}\right),\end{aligned} \qquad [11]$$

where $p_1 = \mathbb{E}_\mathbf{x}[\mathbb{I}\{\mathbf{w}^\mathsf{T}(\mathbf{x}+\mathbf{n}_x) > \theta\}]$ is the probability that the neuron is active, i.e., that $\mathbf{w}^\mathsf{T}(\mathbf{x}+\mathbf{n}_x) > \theta$. Now, since $\mathbf{x}+\mathbf{n}_x$ is Gaussian with mean 0 and covariance $\mathbf{C}_x + \mathbf{C}_{\text{in}}$, we have $\mathbf{w}^\mathsf{T}(\mathbf{x}+\mathbf{n}_x) \sim \mathcal{N}(0, g_0 + \sigma_{\text{in}}^2)$ and

$$p_1 = 1 - \Phi\left(\frac{\theta}{\sqrt{g_0 + \sigma_{\text{in}}^2}}\right) = S\left(\frac{\theta}{\sqrt{g_0 + \sigma_{\text{in}}^2}}\right), \qquad [12]$$

where Φ and S are the cumulative distribution function and the survival function of the standard normal distribution, respectively. Note that if we define $\varsigma^2 \stackrel{\text{def}}{=} g_0 + \sigma_{\text{in}}^2$ to denote the signal variance and $\tilde{\theta} \stackrel{\text{def}}{=} \theta/\varsigma$ to denote the effective threshold, we see that the effective threshold increases when signal variance decreases. We can calculate γ as:

$$\begin{aligned}\gamma^{-1} &= \int_\theta^\infty (y-\theta)\frac{1}{\sqrt{2\pi(g_0+\sigma_{\text{in}}^2)}} e^{-\frac{y^2}{2(g_0+\sigma_{\text{in}}^2)}}\,dy \\ &= \sqrt{\frac{g_0+\sigma_{\text{in}}^2}{2\pi}}\, e^{-\frac{\theta^2}{2(g_0+\sigma_{\text{in}}^2)}} - \theta p_1 \\ &= \varsigma\left(\frac{1}{\sqrt{2\pi\varsigma}} e^{-\frac{\theta^2}{2\varsigma^2}} - \frac{\theta}{\varsigma} S\left(\frac{\theta}{\varsigma}\right)\right) \\ &= \varsigma\left(\varphi(\tilde{\theta}) - \tilde{\theta} S(\tilde{\theta})\right)\end{aligned} \qquad [13]$$

where φ is the probability density function of the standard normal distribution. Substituting this to **Eq 11**, we get:

$$\mathcal{I}_1 = S(\tilde{\theta}) \underbrace{\log\left(1 + \frac{g_0}{\sigma_{\text{in}}^2 + \varsigma^2\sigma_{\text{out}}^2\left(\varphi(\tilde{\theta}) - \tilde{\theta}S(\tilde{\theta})\right)^2}\right)}_{\stackrel{\text{def}}{=} A_1}, \qquad [14]$$

which was used to plot the single-neuron mutual information contributions in **Fig. 4A**.

A single-neuron case with heavy tails.

To investigate the relationship between threshold and outliers in the image distribution, we performed the analysis in **Fig. 4B**, which replaced the Gaussian image distribution with a Student's t-distribution. This allowed us to adjust the heaviness of the tails of the distribution by changing the degrees of freedom from $\nu = 4$ (heavy tails) to $\nu = \infty$ (Gaussian). In this case, a formula similar to **Eq 14** can be derived in a similar manner to **Eq 13**.

More explicitly, if $\mathbf{x} \sim t_\nu(0, \mathbf{C}_x)$, then $\mathbf{w}^\mathsf{T}(\mathbf{x}+\mathbf{n}_x)/\varsigma$ is likewise t_ν-distributed, and we have:

$$p_1 = 1 - \Phi_\nu\left(\frac{\theta}{\varsigma}\right). \qquad [15]$$

where Φ_ν is the cumulative distribution function of the t-distribution. Moreover,

$$\begin{aligned}&\gamma^{-1} \\ &= \int_{\frac{\theta}{\varsigma}}^\infty (\varsigma t - \theta)\frac{\Gamma\left(\frac{\nu+1}{2}\right)}{\sqrt{\nu\pi}\Gamma\left(\frac{\nu}{2}\right)}\left(1+\frac{t^2}{\nu}\right)^{-\frac{\nu+1}{2}} dt \\ &= \frac{\Gamma\left(\frac{\nu+1}{2}\right)}{\sqrt{\nu\pi}\Gamma\left(\frac{\nu}{2}\right)}\frac{\varsigma\nu}{2}\int_{\frac{\theta^2}{\varsigma^2\nu}}^\infty (1+u)^{-\frac{\nu+1}{2}}\,du - \theta p_1 \\ &= \frac{\Gamma\left(\frac{\nu+1}{2}\right)}{\sqrt{\nu\pi}\Gamma\left(\frac{\nu}{2}\right)}\frac{\varsigma\nu}{\nu-1}\left(1+\frac{\theta^2}{\varsigma^2\nu}\right)^{-\frac{\nu-1}{2}} - \theta p_1 \\ &= \varsigma\left(\frac{\nu+\tilde{\theta}^2}{\nu-1}\varphi_\nu\left(\tilde{\theta}\right) - \tilde{\theta}S_\nu\left(\tilde{\theta}\right)\right)\end{aligned} \qquad [16]$$

where φ_ν and S_ν are respectively the probability density function and the survival function of Student's t-distribution with ν degrees of freedom. We see that $\frac{\nu+\tilde{\theta}^2}{\nu-1} \to 1$ as $\nu \to \infty$, in which case **Eq 16** is identical to **Eq 13**. These results can then be substituted into **Eq 11** to derive an expression analogous to **Eq 14**.

Decomposition of the mutual information.

In previous sections, we have considered the encoding properties of a single neuron in isolation. Here, we return to the full optimization objective in **Eq 5**, showing how we can rewrite this as

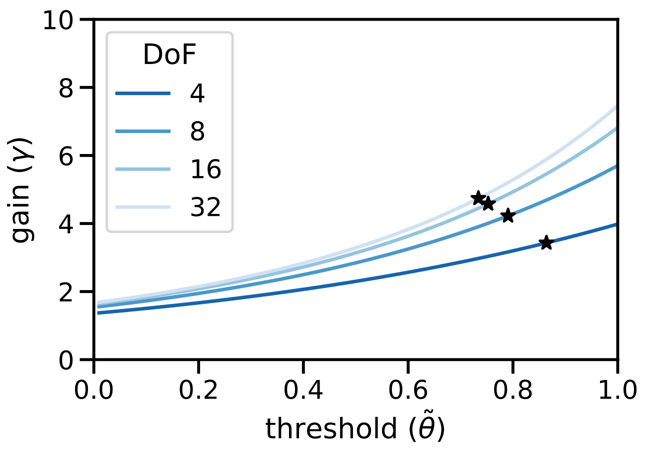

Figure S11. When the input distribution has heavier tails, modeled with Student's t distributions with varying degrees of freedom, the optimal gain decreases with heavier tails. The optimal thresholds are depicted in **Fig. 4B**, and optimal gains are computed using the optimal thresholds and **Eq 16** with ς = 1.249.

a sum of independent, single-neuron contributions plus pairwise (and higher-order) correction terms.

We begin by noting that the expectation in **Eq 5** over images $\mathbf{x}$ involves a sum over patches that only activate subsets of neurons. More specifically, with the hard rectifying nonlinearity assumed in the previous section, we have $\mathbf{G}(\mathbf{x}) = \gamma\mathbb{I}\{\mathbf{w}^\top(\mathbf{x}+\mathbf{n}_x) > \theta\}$ for ON cells and the same for OFF cells with $\theta \to -\theta$. Here, $\mathbb{I}$ is the indicator function, which is 1 when its argument is true and 0 otherwise. Thus, the gain matrix is diagonal, with entries equal to either 0 or γ, and the matrix products involving $\mathbf{G}(\mathbf{x})$ in **Eq 5** are thus low-rank for most $\mathbf{x}$. As a result, we can rewrite the expectation as a sum over log determinants of low-rank matrices, each of which corresponds to a specific collection of active neurons.

For example, consider a three-neuron system, whose activity can be coded as a binary triple, (e.g., 010). Let A be the determinant in **Eq 6**. Letting $p_{\text{(triplet)}}$ denote the probability that each subset is activer over the image set and $A_{\text{(triplet)}}$ be the corresponding determinant ratio, the expectation can be expanded as:

$$\begin{aligned}\mathbb{E}[\log A] = p_{000}\log A_{000} + p_{001}\log A_{001} \\ + p_{010}\log A_{010} + p_{011}\log A_{011} \\ + p_{100}\log A_{100} + p_{101}\log A_{101} \\ + p_{110}\log A_{110} + p_{111}\log A_{111}.\end{aligned} \quad [17]$$

Unfortunately, even for a Gaussian image distribution, calculating p for a specific triple involves a high-dimensional Gaussian integral bounded by a triple of hyperplanes in image space. However, we can rewrite this collection of terms as:

$$\begin{aligned}\mathbb{E}[\log A] &= p_{000}\log A_{000} \\ &+ (p_{100}+p_{110}+p_{101}+p_{111})\log A_{100} \\ &+ (p_{010}+p_{110}+p_{011}+p_{111})\log A_{010} \\ &+ (p_{001}+p_{101}+p_{011}+p_{111})\log A_{001} \\ &+ (p_{110}+p_{111})\log\frac{A_{110}}{A_{100}A_{010}} \\ &+ (p_{101}+p_{111})\log\frac{A_{101}}{A_{100}A_{001}} \\ &+ (p_{011}+p_{111})\log\frac{A_{011}}{A_{010}A_{001}} \\ &+ p_{111}\log\left(A_{111}\frac{A_{100}A_{010}A_{001}}{A_{110}A_{101}A_{011}}\right)\end{aligned} \quad [18]$$

The first term corresponds to the case when no neuron is active, for which $\log A_{000} = 0$. For the next three terms, each of the probabilities in parentheses is a *marginal* probability of a neuron being active. That is, it is equal to p_1 as calculated above. Similarly, the next three terms constitute pairwise corrections to the first set of terms: they involve marginal probabilities of pairwise activation, and their log ratios vanish when the individual neurons are independent (e.g., $A_{110} = A_{100}A_{010}$). Thus, as the distance between a pair of receptive field centers increases beyond the image correlation length scale, these correction terms should vanish.

In our analysis, we focus on the limit in which only first- and second-order interactions are non-negligible. This is an assumption that does not, in fact, hold for natural images (or even our Gaussian images with long-range correlations), though as we shall see, these interactions are sufficient to demonstrate the existence of a phase transition. Moreover, as Figures S5 and S6 show, the same phase transition occurs in simulation when images with long-range correlations are used, suggesting that the principles we identify in the pairwise limit generalize to the full model.

Pairwise interactions: h_2 ***and*** $h_{2'}$***.***
Returning to our 1-D model with N ON and N OFF neurons, we note that, as per **Eq 5** in the main text, we can further decompose the pairwise corrections into two terms, one corresponding to ON-ON and OFF-OFF corrections, the other to ON-OFF pairs. More explicitly, if we denote by a_j and $\bar{a}_j$ the binary variables corresponding to whether the ON cell at location j and the OFF cell at location $j+\phi$ are active, respectively, we can write the mutual information up to pairwise corrections as:

$$2Np_1\log A_1 + \underbrace{N\sum_{j\neq 0}\mathbb{E}_{\mathbf{x}}[a_0 a_j]\log\frac{A_{2j}}{A_1^2}}_{-N(N-1)h_2} + \underbrace{N\sum_{j}\mathbb{E}_{\mathbf{x}}[a_0\bar{a}_j]\log\frac{\bar{A}_{2j}}{A_1^2}}_{-N^2 h_{2'}} \quad [19]$$

where the first term contains $\mathcal{I}_1$ from **Eq 14**, and the next two terms correspond to pairwise interactions between neurons of the same and different polarities, respectively:

$$h_2 \overset{\text{def}}{=} -\frac{1}{N-1}\sum_{j\neq 0}\mathbb{E}_{\mathbf{x}}[a_0 a_j]\log\frac{A_{2j}}{A_1^2} \quad [20]$$

$$h_{2'}(\phi) \overset{\text{def}}{=} -\frac{1}{N}\sum_{j}\mathbb{E}_{\mathbf{x}}[a_0\bar{a}_j]\log\frac{\bar{A}_{2j}}{A_1^2}, \quad [21]$$

Note here that we have used translational invariance to write the pairwise corrections as the interaction between a single ON cell at

0 and all other cells and that there are $\binom{N}{2}$ such pairs. Likewise, since the ON and OFF cells are identical up to a polarity flip, the h_2 term includes both mosaics' self-interactions for a total of $N(N-1)$ such terms. Similarly, there are N^2 ON-OFF interactions, resulting in the differing normalizations above.

Clearly, only the last term in **Eq** 19 is a function of ϕ, and we only need to consider the value ϕ that maximizes $h_{2'}$ to determine whether alignment ($\phi = 0$) or anti-alignment ($\phi = 0.5$) is preferred. We know from **Eq** 11 that

$$A_1 = 1 + \frac{g_0}{\sigma_{\text{in}}^2 + \sigma_{\text{out}}^2\gamma^{-2}}, \quad [22]$$

and we can calculate the two-neuron determinant ratio $\bar{A}_{2j}$ as follows: First define matrices $\mathbf{P}$ and $\mathbf{Q}$ from **Eq** 5 to write

$$A = \frac{\det(\overbrace{\mathbf{GW}^\mathsf{T}\mathbf{C}_x\mathbf{WG}}^{\overset{\text{def}}{=}\mathbf{Q}} + \overbrace{\mathbf{GW}^\mathsf{T}\mathbf{C}_{in}\mathbf{WG} + \mathbf{C}_{out}}^{\overset{\text{def}}{=}\mathbf{P}})}{\det(\underbrace{\mathbf{GW}^\mathsf{T}\mathbf{C}_{in}\mathbf{WG} + \mathbf{C}_{out}}_{\overset{\text{def}}{=}\mathbf{P}})} = \frac{\det(\mathbf{Q}+\mathbf{P})}{\det(\mathbf{P})}, \quad [23]$$

then substitute kernels $\mathbf{w}_0$ and $-\mathbf{w}_j$ (negative because of the opposite polarity):

$$\begin{aligned}\mathbf{P} &= \mathbf{GW}^\mathsf{T}\mathbf{C}_{in}\mathbf{WG} + \mathbf{C}_{out} \\ &= \gamma^2\sigma_{\text{in}}^2\begin{bmatrix}\mathbf{w}_0^\mathsf{T}\mathbf{w}_0 & -\mathbf{w}_0^\mathsf{T}\mathbf{w}_j \\ -\mathbf{w}_j^\mathsf{T}\mathbf{w}_0 & \mathbf{w}_j^\mathsf{T}\mathbf{w}_j\end{bmatrix} + \begin{bmatrix}\sigma_{\text{out}}^2 & 0 \\ 0 & \sigma_{\text{out}}^2\end{bmatrix} \\ &= \begin{bmatrix}\gamma^2\sigma_{\text{in}}^2 + \sigma_{\text{out}}^2 & -\gamma^2\sigma_{\text{in}}^2 f_j \\ -\gamma^2\sigma_{\text{in}}^2 f_j & \gamma^2\sigma_{\text{in}}^2 + \sigma_{\text{out}}^2\end{bmatrix}\end{aligned} \quad [24]$$

and

$$\begin{aligned}\mathbf{Q} &= \mathbf{GW}^\mathsf{T}\mathbf{C}_x\mathbf{WG} \\ &= \gamma^2\begin{bmatrix}\mathbf{w}_0^\mathsf{T}\mathbf{C}_x\mathbf{w}_0 & -\mathbf{w}_0^\mathsf{T}\mathbf{C}_x\mathbf{w}_j \\ -\mathbf{w}_j^\mathsf{T}\mathbf{C}_x\mathbf{w}_0 & \mathbf{w}_j^\mathsf{T}\mathbf{C}_x\mathbf{w}_j\end{bmatrix} = \gamma^2\begin{bmatrix}g_0 & -g_j \\ -g_j & g_0\end{bmatrix},\end{aligned} \quad [25]$$

where we define $g_j \overset{\text{def}}{=} g(|\bar{z}_j|)$, $f_j \overset{\text{def}}{=} f(|\bar{z}_j|)$, and thus $f_0 = 1$. We then have

$$\det \mathbf{P} = (\gamma^2\sigma_{\text{in}}^2 + \sigma_{\text{out}}^2)^2\left[1 - \left(\frac{\gamma^2\sigma_{\text{in}}^2 f_j}{\gamma^2\sigma_{\text{in}}^2 + \sigma_{\text{out}}^2}\right)^2\right] \quad [26]$$

and

$$\begin{aligned}&\det(\mathbf{P}+\mathbf{Q}) \\ &= \det\begin{bmatrix}\gamma^2(g_0 + \sigma_{\text{in}}^2) + \sigma_{\text{out}}^2 & -\gamma^2(g_j + \sigma_{\text{in}}^2 f_j) \\ -\gamma^2(g_j + \sigma_{\text{in}}^2 f_j) & \gamma^2(g_0 + \sigma_{\text{in}}^2) + \sigma_{\text{out}}^2\end{bmatrix} \\ &= (\gamma^2(g_0 + \sigma_{\text{in}}^2) + \sigma_{\text{out}}^2)^2\left[1 - \left(\frac{\gamma^2(g_j + \sigma_{\text{in}}^2 f_j)}{\gamma^2(g_0 + \sigma_{\text{in}}^2) + \sigma_{\text{out}}^2}\right)^2\right].\end{aligned} \quad [27]$$

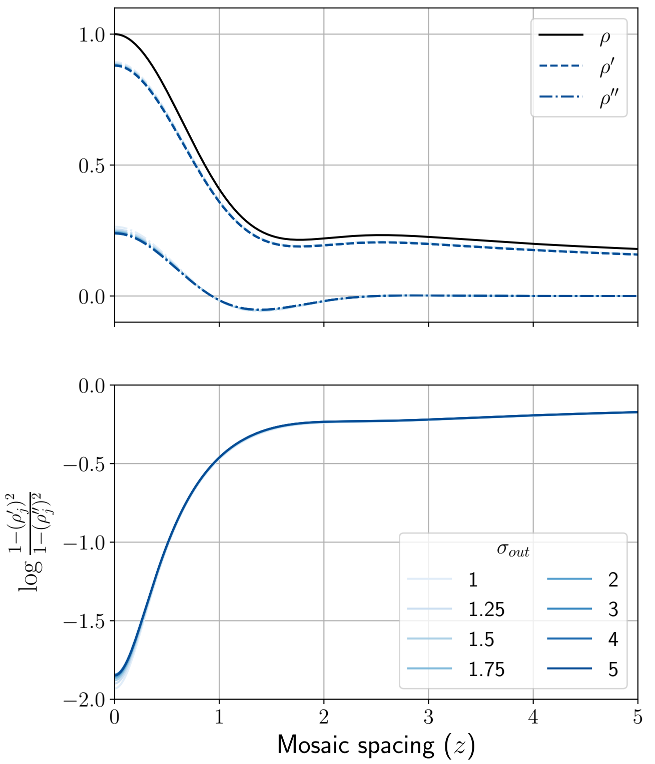

Figure S12. Plots of the correlation-like quantities ρ, ρ', and ρ'' as defined in **Eqs** 32 and 28 and the log ratio $\log \frac{\bar{A}_{2j}}{A_1^2} = \log \frac{1-(\rho')^2}{1-(\rho'')^2}$, with respect to color-coded σ_{out} values. These depend very weakly on σ_{out}, suggesting the $\mathbb{E}_{\mathbf{x}}[a_0\bar{a}_j]$ term in **Eq** 21 plays a more crucial role in the phase transition.

Collecting these with the expression for A_1 in **Eq** 22,

$$\begin{aligned}\frac{\bar{A}_{2j}}{A_1^2} &= \frac{\dfrac{\det(\mathbf{Q}+\mathbf{P})}{\det(\mathbf{P})}}{\left(1 + \dfrac{g_0}{\sigma_{\text{in}}^2 + \sigma_{\text{out}}^2\gamma^{-2}}\right)^2} \\ &= \frac{1 - \Bigg(\overbrace{\dfrac{g_j + \sigma_{\text{in}}^2 f_j}{g_0 + \sigma_{\text{in}}^2 + \sigma_{\text{out}}^2\gamma^{-2}}}^{\overset{\text{def}}{=}\rho_j'}\Bigg)^2}{1 - \Bigg(\underbrace{\dfrac{\sigma_{\text{in}}^2 f_j}{\sigma_{\text{in}}^2 + \sigma_{\text{out}}^2\gamma^{-2}}}_{\overset{\text{def}}{=}\rho_j''}\Bigg)^2} = \frac{1 - (\rho_j')^2}{1 - (\rho_j'')^2}\end{aligned} \quad [28]$$

Note that since $f_j \leq 1$ by definition and $g_j \leq g_0$ for most reasonable image distributions, both terms in brackets are less than 1. Also, as $j \to \infty$, we have $g_j, f_j \to 0$, so the term vanishes asymptotically. In fact, f_j vanishes very quickly (as soon as the receptive fields have negligible overlap), while g_j diminishes much more slowly, since $\mathbf{C}_x$ contains long-range correlations. Note that f_j and g_j are the only terms here that depend on ϕ. More importantly, this term only varies weakly with σ_{out} as shown in **Fig. S12**, suggesting it does not play a crucial role in the phase transition.

Now, as for the coactivation probability $\mathbb{E}_{\mathbf{x}}[a_0\bar{a}_j]$, we consider $y_0 = \mathbf{w}_0^\mathsf{T}(\mathbf{x} + \mathbf{n}_x)$ and $y_j = -\mathbf{w}_j^\mathsf{T}(\mathbf{x} + \mathbf{n}_x)$ whose joint distribution

follows

$$(y_0, y_j) \sim \mathcal{N}\left(\mathbf{0}, \begin{bmatrix} g_0 + \sigma_{\text{in}}^2 & -g_j - \sigma_{\text{in}}^2 f_j \\ -g_j - \sigma_{\text{in}}^2 f_j & g_0 + \sigma_{\text{in}}^2 \end{bmatrix}\right), \quad [29]$$

which means that $\mathbb{E}_{\mathbf{x}}[a_0 \bar{a}_j]$ can be calculated via a Gaussian integral over the region $y_0 > \theta$ and $y_j > \theta$:

$$\begin{aligned} \mathbb{E}[a_0\bar{a}_j] &= \int_\theta^\infty \int_\theta^\infty p(y_0, y_j)\, dy_0\, dy_j \\ &= \int_\theta^\infty \frac{1}{\sqrt{2\pi}} e^{-\frac{y^2}{2}} \left(1 - \Phi\left(\frac{\tilde{\theta} - \rho_j y}{\sqrt{1-\rho_j^2}}\right)\right) dy \\ &\overset{\text{def}}{=} p_2(\tilde{\theta}, \rho_j) \end{aligned} \quad [30]$$

where $\tilde{\theta}$ and ρ_j are defined as

$$\tilde{\theta} = \theta / \sqrt{g_0 + \sigma_{\text{in}}^2} \quad [31]$$

$$\rho_j \overset{\text{def}}{=} \frac{g_j + \sigma_{\text{in}}^2 f_j}{g_0 + \sigma_{\text{in}}^2}. \quad [32]$$

Putting all this together, we can write $h_{2'}$ as

$$\begin{aligned} h_{2'}(\phi) &= -\frac{1}{N} \sum_j p_2(\tilde{\theta}, \rho_j) \log \frac{1 - (\rho_j')^2}{1 - (\rho_j'')^2} \\ &= -\frac{1}{N^2} \sum_{i,j} p_2(d_{ij}) \log R(d_{ij}), \end{aligned} \quad [33]$$

where again, the dependence on ϕ is solely through $f_j = f(|j+\phi|)$ and $g_j = g(|j+\phi|)$ and we have used translation invariance to rewrite these arguments as d_{ij}, the distance between ON-OFF pairs. Finally, since $f_j \leq 1$ by definition, $\rho_j > \rho_j'$ always, and $\rho_j' > \rho_j'' \iff \frac{g_j}{g_0} > \rho_j''$ (which holds empirically with our image distribution and optimized kernels), we have $0 < \frac{1-(\rho_j')^2}{1-(\rho_j'')^2} < 1$ and thus $\mathcal{I}_{2'} \leq 0$.

For the results plotted in **Fig. 5A**, we numerically integrated **Eq 30** to calculate p_2 and used kernels and covariance parameters taken from a trained one-shape model, shown in **Fig. S10**, to calculate $\mathbf{C}_x$, f_j, and g_j. To make these quantities comparable as σ_{out} increases (and thus p_2 decreases), **Fig. 5A** plots the normalized contribution $Nh_{2'}/p_1^2$.

C. Redundancy in aligned and independent ON-OFF pairs.

We have argued in **Fig. 4D-E** that redundancy is reduced by aligning a single OFF cell (in the case of low θ) and anti-aligning multiple OFF neighbors (for high θ) inside a "reduced redundancy" region of small p_2 around each ON cell. However, this characterization may be objected to on the grounds that, in fact, aligned response fields are highly redundant, since the activation of one by a stimulus excludes activation of the other.

In a limited sense, this is true. Consider replacing our monotonically increasing nonlinearity h with a simple threshold nonlinearity, such that neurons fire at a constant rate when active, similar to (3). In this case, we can calculate for a single neuron with $\theta = 0$

$$H(\mathbf{X}|\mathbf{R}) = -p(0)\log p(0) - p(1)\log p(1) = \log 2 = 1 \text{ bit} \quad [34]$$

since the neuron is expected to be active half the time. Clearly, for two independent cells (e.g., those with receptive field centers separated by a distance much larger than the image correlation scale), we then expect 2 bits of conditional entropy. However, for an aligned ON-OFF pair, we would have

$$H(\mathbf{X}|\mathbf{R}) = -p(0,1)\log p(0,1) - p(1,0)\log p(1,0) = \log 2, \quad [35]$$

just as before. Thus, when each neuron has a capacity of one bit, two aligned receptive fields are completely redundant.

However, the assumption of a graded firing rate conveys additional information about the stimulus beyond its sign, and this information is not fully shared even by aligned receptive fields. For instance, in the case of a single neuron, we have from **Eqs 14** and **22**

$$H(\mathbf{X}|\mathbf{R}) \approx \frac{1}{2}\log\left(1 + \frac{g_0}{\sigma_{\text{in}}^2 + \sigma_{\text{out}}^2 \frac{g_0}{2\pi}}\right) \approx 0.5 \log 5.7 \quad [36]$$

for $\theta = 0$, $\sigma_{\text{in}} = 0.4$, $\sigma_{\text{out}} = 1$, $g_0 \approx 3.8$. However, this is *differential* entropy. To relate this to the answer in the 1-bit case, we need to consider a quantization of the signal. If we assume that the signal can be quantized using 1 bit for its sign and n bits for its magnitude, we have, for n sufficiently large (4)

$$H(\mathbf{X}^\Delta|\mathbf{R}) \approx \tilde{H}(\mathbf{X}|\mathbf{R}) + (n+1)\log 2 \quad [37]$$

where $\mathbf{X}^\Delta$ is the quantized variable, and we denote the differential entropy by $\tilde{H}$.

From this result, it is clear that, while far-separated receptive fields encode precisely $2H(\mathbf{X}^\Delta|\mathbf{R})$, perfectly aligned receptive fields encode only $2H(\mathbf{X}^\Delta|\mathbf{R}) - \log 2$, where the correction is due to the shared sign bit. Thus, while it is correct that aligned receptive fields do exhibit some redundancy (1 bit for $\theta = 0$, less as θ increases), this is typically much smaller than the information about stimulus magnitude. More importantly, when considering how to align ON and OFF cells, informational losses due to shared sign bits are dwarfed by the size of $N^2 h_{2'}$ for neighbors located outside the gray zone in **Fig. 4A**.

References

1. Doi E, Lewicki MS (2007) A theory of retinal population coding in *Advances in neural information processing systems*. pp. 353–360.
2. Karklin Y, Simoncelli EP (2011) Efficient coding of natural images with a population of noisy linear-nonlinear neurons in *Advances in neural information processing systems*. pp. 999–1007.
3. Gjorgjieva J, Sompolinsky H, Meister M (2014) Benefits of pathway splitting in sensory coding. *Journal of Neuroscience* 34(36):12127–12144.
4. Cover TM (1999) *Elements of information theory*. (John Wiley & Sons).
